# Chemo-enzymatic production of base-modified ATP analogues for polyadenylation of RNA†

**DOI:** 10.1039/d4sc03769c

**Published:** 2024-07-16

**Authors:** Rachel M. Mitton-Fry, Jannik Eschenbach, Helena Schepers, René Rasche, Mehmet Erguven, Daniel Kümmel, Andrea Rentmeister, Nicolas V. Cornelissen

**Affiliations:** a Institute of Biochemistry, University of Münster Corrensstr. 36 D-48149 Münster Germany mittonfryr@denison.edu andrea.rentmeister@cup.lmu.de cornelissen@uni-muenster.de; b Department of Chemistry and Biochemistry, Denison University 100 W. College St., Granville Ohio 43023 USA; c Institute of Chemical Epigenetics, Ludwig-Maximilians-University Munich Butenandtstr. 5-13, Haus F D-81377 Munich Germany; d Cells in Motion Interfaculty Centre, University of Münster Waldeyerstraße 15 D-48149 Münster Germany

## Abstract

Base-modified adenosine-5′-triphosphate (ATP) analogues are highly sought after as building blocks for mRNAs and non-coding RNAs, for genetic code expansion or as inhibitors. Current synthetic strategies lack efficient and robust 5′-triphosphorylation of adenosine derivatives or rely on costly phosphorylation reagents. Here, we combine the efficient organic synthesis of base-modified AMP analogues with enzymatic phosphorylation by a promiscuous polyphosphate kinase 2 class III from an unclassified *Erysipelotrichaceae* bacterium (EbPPK2) to generate a panel of C2-, N^6^-, or C8-modified ATP analogues. These can be incorporated into RNA using template independent poly(A) polymerase. C2-halogenated ATP analogues were incorporated best, with incorporations of 300 to >1000 nucleotides forming hypermodified poly(A) tails.

## Abstract 2

We demonstrate the efficient chemo-enzymatic synthesis of base-modified adenosine-5′-triphosphate (ATP) analogues. These can be incorporated into RNA using poly(A) polymerase to produce RNAs with hypermodified poly(A) tails.

## Graphical abstract

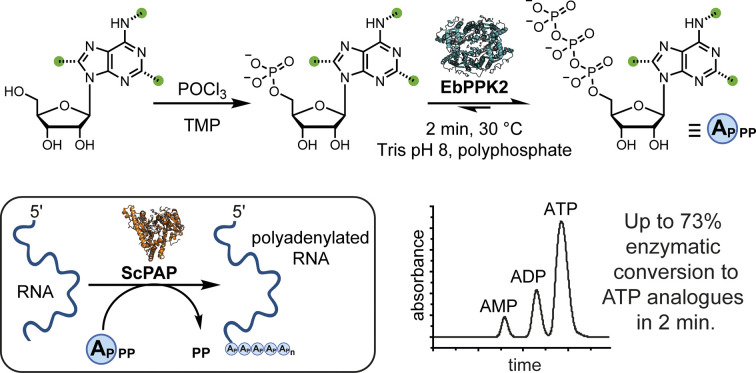

## Introduction

The production of mRNA therapeutics requires modified nucleoside-5′-triphosphates (NTPs), *e.g.*, pseudouridine (Ψ) and N1-methylpseudouridine (m^1^Ψ) replacing uridine (U), which increase the stability of mRNA and lower the resulting immune response.^1^ In contrast to replacing nucleotides in the entire mRNA,^2^ post-transcriptional modification of the poly(A) tail has been shown to improve protein production in cells independent of the encoded protein of interest.^3^ Additionally, base-modified nucleotides play critical regulatory roles in a variety of noncoding RNAs, and incorporation of non-natural nucleotides allows the study or manipulation of RNA localization and function.^4^

In spite of recent improvements in NTP synthesis,^5^ low yields in the last 5′-triphosphorylation steps (typically 4–33%) limit the number of base-modified NTPs available.^6^ For instance, the synthesis of base-modified ATP analogues is considered “troublesome”^7^ and often low yielding (8–30%)^8^ with few exceptions,^9^ rendering it an underexplored chemical space.

While chemical 5′-monophosphorylation of adenosine is known to proceed efficiently (>90%),^10^ enzymatic strategies have emerged as viable alternatives for 5′-triphosphorylation.^11^

Polyphosphate kinase family 2 (PPK2) enzymes are particularly attractive, as they use inexpensive and non-toxic Graham's salt (sodium polyphosphate) as a cofactor to phosphorylate AMP or ADP and derivatives thereof.^12^ For example, base-modified ATP analogues have been prepared using a two enzyme PPK2-I/PPK2-II mixture.^11*a*^ Recently, Tavanti *et al.* screened 92 PPKs and identified a PPK2 class III enzyme (PPK2-III) from an unclassified *Erysipelotrichaceae* bacterium (EbPPK2) which served as a robust and efficient ATP recycling catalyst.^13^ As observed for other PPK2-III enzymes,^14^ EbPPK2 accepts Graham's salt as a phosphate source and efficiently catalyses production of both ADP and ATP starting from AMP. The reported equilibria of AMP (∼3%), ADP (∼27%) and ATP (∼70%) are consistent with thermodynamic calculations for other PPK2 enzymes.^15^

In this work, base-modified adenosine analogues were phosphorylated chemically, and the resulting AMP analogues were converted to the corresponding ATP analogues by EbPPK2. We applied the chemo-enzymatically generated ATP analogues to *in vitro* reactions with poly(A) polymerase (PAP) to prepare 30-mer RNAs with hypermodified poly(A) tails.

## Results and discussion

We generated a structural model of EbPKK2 using AlphaFold2 ^16^ in order to examine its nucleotide-binding pocket. As other PPK2-III enzymes are known to form tetrameric complexes,^12*c*^ we used four subunits to generate a multimeric model.^17^

**Fig. 1:**
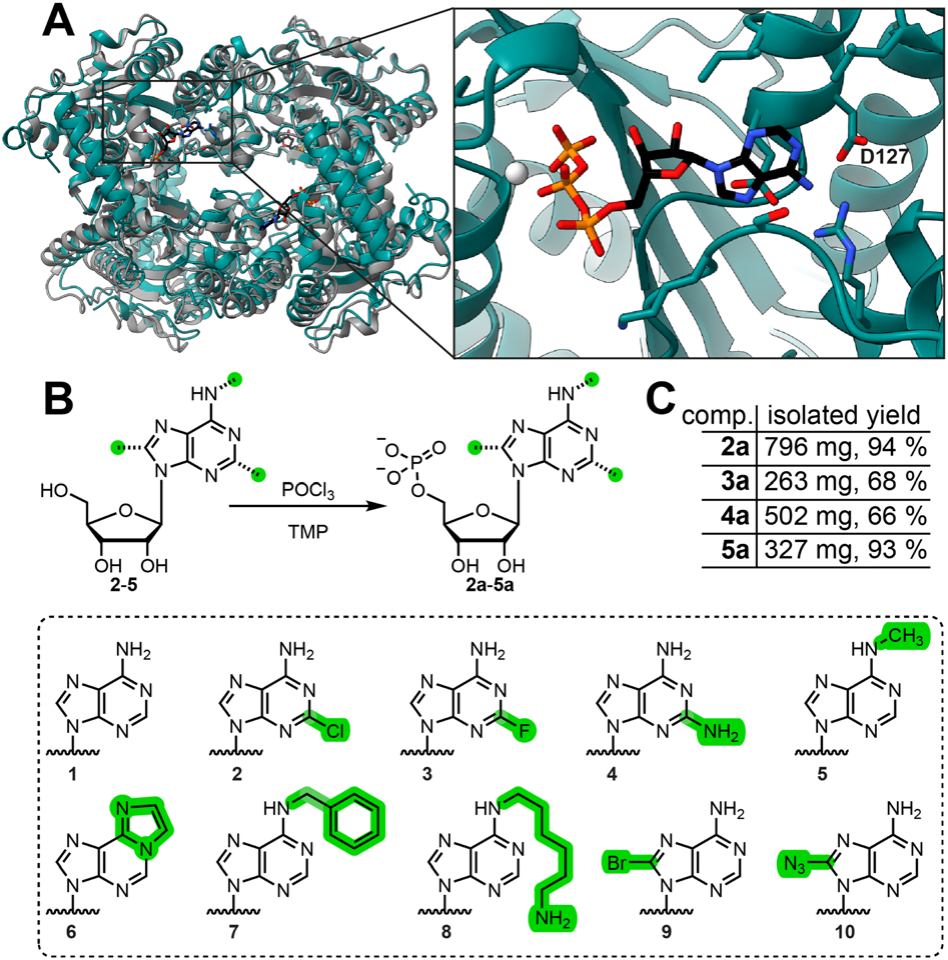
Model of EbPPK2 and substrate panel of AMP analogues. (A) AlphaFold2 model of tetrameric EbPPK2 (teal), overlaid with the tetrameric PPK2 from *Meiothermus ruber* (gray) bound to ATP (PDB: 5LD1). Protein tetramers aligned with a root mean square deviation (RMSD) of 0.8 Å over 231 C_α_ atom pairs. Inset shows the predicted active site from a single subunit of EbPPK2, with all sidechains found within 4 Å of the nucleobase of ATP represented as sticks. D127 (labelled) coordinates the N^6^-position of the base. Alignment, RMSD calculation and figure made using Chimera.^18^ (B) Scheme of the chemical synthesis of base-modified AMP analogues (top) and the nucleobase portion of substrates tested for EbPPK2 (bottom). Green highlighting represents potential modifications, where H in all positions corresponds to natural adenosine 1. (C) Isolated yields of the base-modified AMP analogues 2a–5a.

The core of the structure was modelled with very high confidence, while the two lid helices of the nucleotide-binding pocket have a slightly lower confidence, suggesting a certain degree of conformational flexibility (Fig. S1†). Our model aligned closely to the crystal structure of a class III PPK2 enzyme from *Meiothermus ruber* bound to ATP (MrPPK2, PDB: 5LD1) (Fig. 1A).^12*c*^ Thus, we inserted the nucleotide from the MrPPK2 structure into our predicted EbPKK2 model and used the YASARA force field minimization server^19^ to improve the accuracy of the nucleotide-binding mode. Analyzing the nucleobase-binding pocket (Fig. 1A, inset), we observed that the C2- and C8-positions of the purine base are not involved in tight contacts with the protein, such that modifications might have sufficient space to be tolerated. Similarly, as the N^6^-position is coordinated by a single aspartate residue (D127), N^6^-modifications were also expected to have the potential to be tolerated.

Therefore, in addition to natural AMP (1a), we synthesized AMP analogues bearing modifications at the C2- and N^6^-positions (2a–5a) using the common procedure reported by Yoshikawa *et al.* in 1967 ^10^ (Fig. 1B). The compounds were purified using C_18_-flash chromatography (Fig. S2†) and obtained as sodium salts after ion exchange with high yield (Fig. 1C). Identity was confirmed by mass spectrometry and NMR (Fig. S3–S18†). This panel was complemented with commercially available base-modified AMP analogues (6a–10a) to include further modifications at the N^6^- and C8-positions to gain insight into the promiscuity of EbPPK2.

**Fig. 2:**
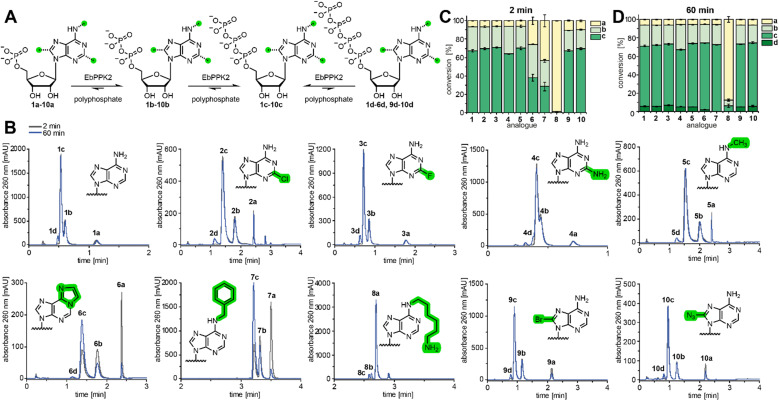
Substrate scope of EbPPK2. (A) Scheme of the EbPPK2-catalysed reactions using polyphosphate. AMP analogues 1a–10a are converted to ADP analogues 1b–10b and ATP analogues 1c–10c until an equilibrium is approached. After 60 min the corresponding tetraphosphates (d) are found for some analogues. (B) Representative UV-chromatograms (260 nm) of the LC-MS analysis of the EbPPK2 reactions using 1a–10a after 2 min and 60 min. Conditions: 1 mM AMP analogue (1a–10a), 6.1 g L^−1^ sodium polyphosphate, 4 µM EbPPK2 incubated for 2 min or 60 min at 30 °C in a total volume of 20 µL. Buffer: 20 mM Tris pH = 8, 20 mM MgCl_2_. The polyphosphate used had an average chain length of 11 phosphate units, according to ^31^P NMR analysis (Fig. S24†), making the phosphate chain concentration approximately 5 mM. (C) and (D) Conversions of the EbPPK2 reactions after 2 min (C) and 60 min (D) as average of three independent experiments with standard deviation (SD).

EbPPK2 was expressed in *E. coli* BL21(DE3) cells, yielding 125 mg of soluble protein per litre of culture after NiNTA purification (Fig. S19 and S20†). Gel filtration chromatography confirmed that EbPPK2 formed a tetramer in solution (Fig. S21†). Thermal stability of EbPPK2 was determined in different buffer conditions, and melting temperatures (*T*_m_) of up to 54 °C were measured (Fig. S22 and S23†). The activity of EbPPK2 was first confirmed using the natural substrate 1a (Fig. 2).

The reaction was performed under mild conditions, with incubation at 30 °C and pH 8. Aliquots were analysed by LC-MS after 2 min and 60 min incubation. The UV chromatogram at 260 nm (Fig. 2B) indicated that the equilibrium of 6% AMP (1a), 26% ADP (1b) and 67% ATP (1c) was approached in only 2 min, with minor conversion to the tetraphosphate (1d) after 1 hour.

Enzymatic reactions with AMP analogues 2a–10a were then performed and analysed (Fig. 2B–D). All UV signals could be unambiguously assigned to the expected [M + H]^+^ ion in ESI-positive mode (Fig. S25–S44†). To our delight, EbPPK2 accepted a broad range of C2-, N^6^-, and C8-modified analogues.

The C2-modified AMP analogues were tolerated well, as 2-chloro-AMP (2a), 2-fluoro-AMP (3a) and 2-amino-AMP (4a) were converted to 70% (2c), 71% (3c) and 64% (4c) of the corresponding ATP analogue in 2 min, respectively. N^6^-modifications led to more variable effects, particularly with respect to the kinetics of the reaction. N^6^-methyl-AMP (5a) gave 70% of the corresponding triphosphate (5c) in 2 min. Reactions using the larger N^6^-modifications etheno-AMP (6a) and N^6^-benzyl-AMP (7a) proceeded more slowly, with only 38% of 6c and 28% of 7c in 2 min, respectively. However, conversions increased to 72% of 6c and 73% of 7c after 60 min, respectively. N^6^-aminohexyl-AMP (8a) illustrates the limit of promiscuity of EbPPK2. In 2 min, only 1% conversion to the corresponding diphosphate (8b) was found, while 8c was not detected. After 60 min, 6% conversion to 8b and 6% to 8c was observed. The C8-modified analogues were also tolerated well, with 8-bromo-AMP (9a) and 8-azido-AMP (10a) giving conversions of 67% (9c) and 70% (10c) in 2 min, respectively. Interestingly, the percent AMP remaining in the C8-modified reactions was higher than observed in other reactions after 2 minutes (∼10% *vs.* ∼6%). After 60 min, the percent AMP in these was reduced to 6%, with concomitant increase of the tetraphosphate product.

**Fig. 3:**
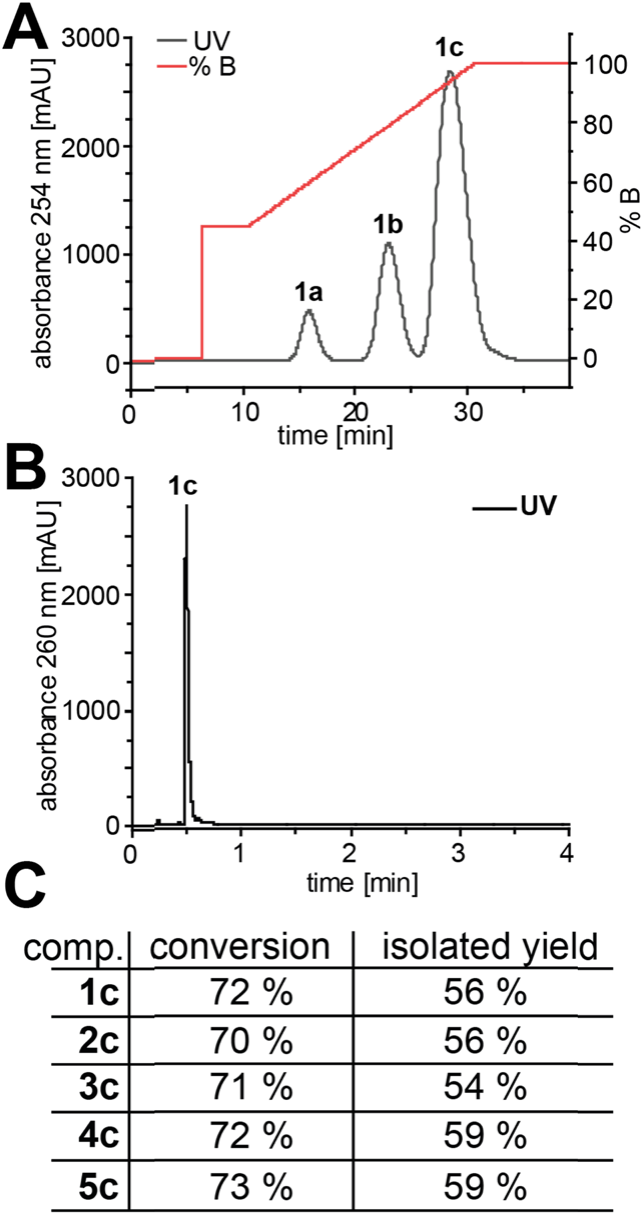
Purification and purity of preparative EbPPK2 reactions (10 µmol). (A) Chromatogram of the anion exchange chromatography exemplified for 1c (for 2c–5c see Fig. S45†). Performed on an ÄKTA purifier. Buffer A: ddH_2_O; buffer B: 100 mM NaClO_4_, pH 4.2. (B) Chromatogram of the LC-MS analysis of ATP (1c). The corresponding chromatograms and EIC for 1c–5c can be found in Fig. S46–S50.† (C) Conversion and isolated yield for 1c–5c.

We then considered the utility of this process for producing sufficient quantities of modified ATP analogues to purify for further use. Therefore, we chose AMP (1a) and analogues 2a–5a for preparative (10 µmol) EbPPK2 reactions. Anion-exchange chromatography allowed excellent resolution of products, indicating 70–73% conversions to ATP analogues 1c–5c (Fig. 3A and S45†). Precipitation of the ATP analogues with acetone resulted in isolated yields of 54–59% with excellent purity (Fig. 3B, C and S46–S50†). Isolated yields were determined *via* UV-absorbance, and the purity was examined *via* LC-MS. The separation observed suggested that this method would also allow purification of ADP analogues for other applications.

**Fig. 4:**
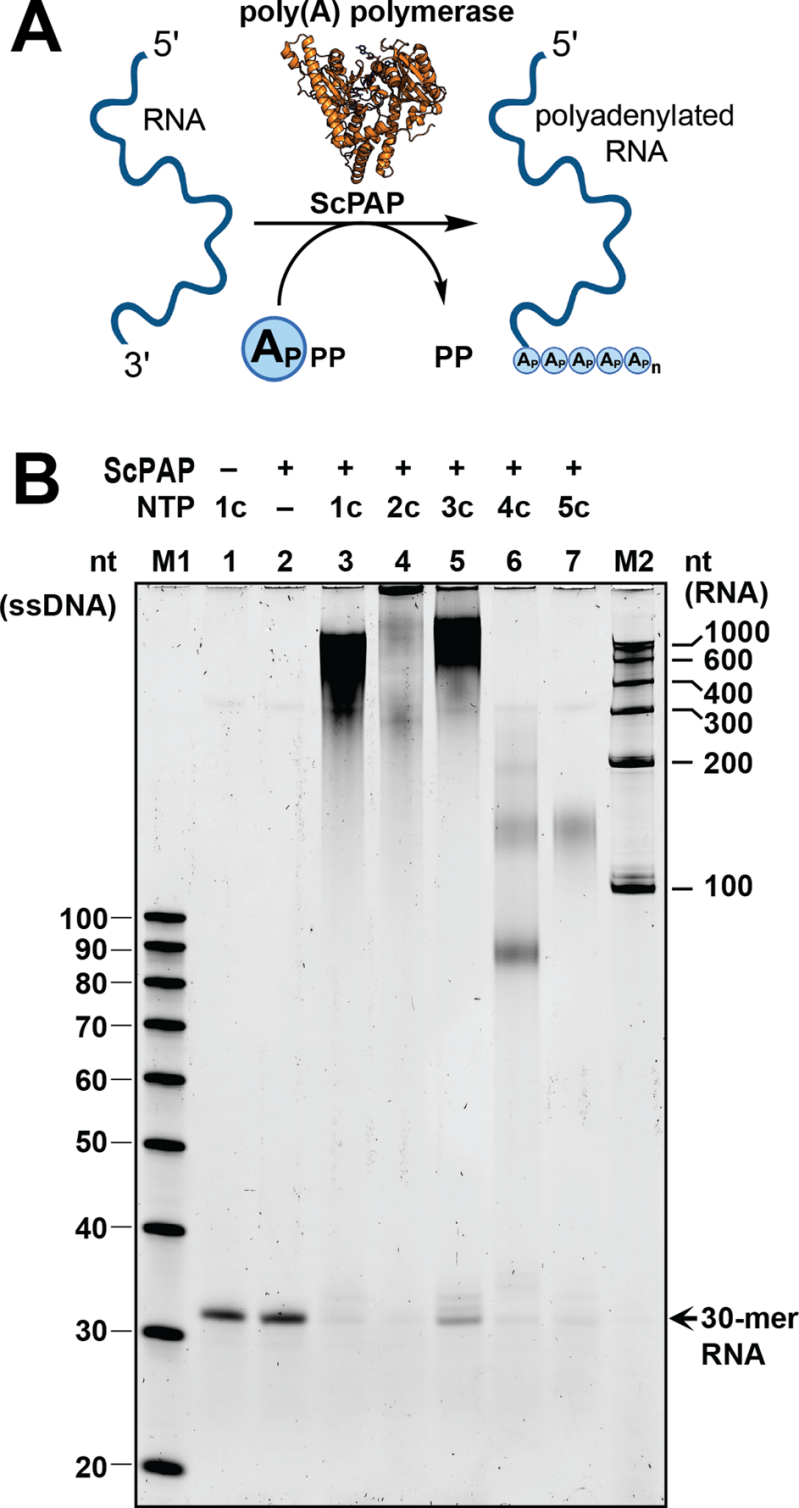
Base-modified ATP analogues can be incorporated into RNA poly(A) tails using yeast poly(A) polymerase (ScPAP). (A) Scheme for uniform incorporation of ATP analogues. Created with http://biorender.com/. (B) 10% denaturing urea polyacrylamide gel (representative) of the polyadenylation reactions using ScPAP and compounds 1c–5c. Replicates shown in Fig. S51.† RNA separation was performed for 1 h at 25 W, gel stained with SYBR Gold, and results visualised with a Typhoon scanner. M1: ssDNA Oligo Length standard 20/100 (Integrated DNA Technologies); M2: RiboRuler RNA Ladder Low Range (Thermo Fisher Scientific).

Poly(A) polymerase from *Saccharomyces cerevisiae* (ScPAP) can be used to incorporate ATP analogues at the 3′ end of RNA in a template-independent fashion, but it does not appear to tolerate C8-modifications.^20^ Therefore, we tested the incorporation of purified analogues 1c-5c into 30-mer RNA using ScPAP (Fig. 4A). Under the conditions tested, ATP (1c) led to a poly(A) tail length distribution of 300–1000 nt (Fig. 4B), underscoring the quality of our ATP preparation. To our surprise, the C2-halogenated ATP analogues 2c and 3c were not only incorporated, but resulted in longer RNAs with distributions of 300 to >1000 nt and 400 to >1000 nt, respectively. The C2-amino-modified analogue 4c led to poly(A) tails distributed around 90 nt and 120 nt. Finally, the N^6^-modified analogue 5c was incorporated with a most common distribution around 125 nt. These results show that all the tested ATP analogues (1c–5c) can be incorporated by ScPAP, leading to hypermodified poly(A) tails of 90 to more than 1000 nt.

## Conclusion

Here, we present a chemo-enzymatic strategy for the synthesis of base-modified ATP analogues. The 5′-monophosphorylation of adenosine analogues is performed by classical chemical synthesis, and the consecutive 5′-triphosphorylation is achieved by a single enzyme, EbPPK2, a polyphosphate kinase 2 class III enzyme. In comparison to other PPKs, the fast catalysis of EbPPK2 (5 s^−1^)^13*a*^ at mild conditions allow the reaction to approach the equilibrium in 2 min at 30 °C in Tris buffer, pH 8 for most of the analogues tested. At analytical scale, conversions of 64–71% were achieved in 2 min for 1c–5c, 9c, and 10c, while analogues 6c and 7c reached 72–73% conversion in 60 min. At preparative scale (10 µmol), 70–73% conversion to ATP analogues (1c–5c) was found, all of which could be purified and then incorporated into RNA using poly(A) polymerase. Next to ATP (1c), the C2-halogenated analogues 2c and 3c were incorporated best (>300 nt). Our chemo-enzymatic approach will help to further explore the chemical space of base-modified ATP analogues, currently limited by unreliable and inefficient 5′-phosphorylation strategies. This will benefit various areas of research, such as genetic code expansion, nucleotide-based inhibitors, RNA labelling and RNA therapeutics. Furthermore, incorporation into RNA by poly(A) polymerases paves the way for mRNAs with hypermodified poly(A) tails.

## Data availability

The data supporting this article have been included as part of the ESI.†

## Author contributions

R. M. M.-F., A. R and N. V. C. conceived the project. R. M. M.-F., J. E., H. S., R. R., M. E. and N. V. C. designed and performed experiments. R. R. and D. K. performed the structure analysis. All authors discussed the results. R. M. M.-F. and N. V. C. wrote the manuscript. All authors discussed and agreed on the final version.

## Conflicts of interest

The authors declare no competing interests.

## Supplementary Material

SC-015-D4SC03769C-s001
