# Supplementary material for: Chemo-enzymatic production of base-modified ATP analogues for polyadenylation of RNA
Source: Chem Sci. 2024 Jul 16;15(32):13068–73. doi: 10.1039/d4sc03769c (PMC11322958; doi:10.1039/d4sc03769c)
Supplement: SC-015-D4SC03769C-s001 [file SC-015-D4SC03769C-s001.pdf]

Electronic Supplementary Material (ESI) for Chemical Science.
This journal is © The Royal Society of Chemistry 2024

Supporting Information

Chemo-enzymatic production of base-modified ATP analogues for polyadenylation of RNA

Rachel M. Mitton-Fry[a,b,*], Jannik Eschenbach[a], Helena Schepers[a], René Rasche[a], Mehmet Erguven[a,c,d], Daniel Kümmel[a,d], Andrea Rentmeister[a,c,d,*], and Nicolas V. Cornelissen[a,*]

[a] Institute of Biochemistry, University of Münster, Corrensstr. 36, D-48149 Münster, Germany.

[b] Department of Chemistry and Biochemistry, Denison University, 100 West College Street Granville, Ohio 43023, USA.

[c] Current address: Institute of Chemical Epigenetics, Ludwig-Maximilians-University Munich, Butenandtstr. 5-13, Haus F, D-81377 Munich, Germany.

[d] Cells in Motion Interfaculty Centre, University of Münster, Waldeyerstraße 15, D-48149 Münster, Germany.

*** E-mail:** mittonfryr@denison.edu, andrea.rentmeister@cup.lmu.de, cornelissen@uni-muenster.de

Table of contents

Materials and methods 4

Extinction coefficients 4

HRMS 4

NMR 4

LC-MS 5

Synthesis of AMP analogues 2a-5a 6

Synthesis of 2-chloroadenosine-5′-monophosphate (2a) 7

Synthesis of 2-fluoroadenosine-5′-monophosphate (3a) 7

Synthesis of 2-aminoadenosine-5′-monophosphate (4a) 8

Synthesis of N^6-methyladenosine-5′-monophosphate (5a) 8

Molecular Modelling 9

EbPPK2 construct: 9

EbPPK2 expression and purification 10

EbPPK2 characterization 11

EbPPK2 analytical scale reactions 11

EbPPK2 preparative scale reactions 12

Purification of ATP analogues 12

Polyadenylation of RNA 13

Supplementary figures 14

Alphafold Structure Predictions 14

Preparative HPLC purification of 2a-5a 15

FTMS spectra of 2a-5a 16

NMR spectra of AMP analogues (2a-5a) 18

Purification of EbPPK2 24

Multimer determination by gel filtration 25

Thermal shift assay 25

LC-DAD-Q-MS analysis of enzymatic reactions (analytical scale) 33

Purification of preparative EbPPK2 reactions.. 38

LC-DAD-Q-MS analysis of purified ATP analogues (1c – 5c).......................... 39

Denaturing urea polyacrylamide gels of poly(A) polymerase reactions........ 42

Materials and methods

All chemicals and reagents were purchased from Sigma-Aldrich, Acros Organics, VWR and Jena Biosciences and were used without further purification unless otherwise stated. As polyphosphate source, Graham´s salt (Sigma-Aldrich 305553) was used.

Extinction coefficients

Nucleotide concentrations were measured via UV absorbance using extinction coefficients indicated by Jena Biosciences, as follows:

Nucleotide	Extinction coeff., mM^{-1} cm^{-1}	Wavelength, nm
ATP	15.1	260
N^6-Me ATP	18.5	265
2-Cl ATP*	15.7	265
2-F ATP	14.3	261
2-NH_2 ATP	9.89	280

*As listed for 2-Cl-dATP

HRMS

HRMS were measured on an Orbitrap Exploris™ 120 mass spectrometer. Samples were dissolved in MeOH and ionized by electrospray ionization.

NMR

NMR spectra were measured at 299 K on a Bruker Neo 400 spectrometer. The chemical shifts (δ) were reported in ppm relative to deuterated solvents as internal standard (D_2O = 4.79 ppm).

LC-MS

LC-DAD-Q-MS were performed on an Agilent 1260 Infinity II Prime equipped with a 1260 high sensitivity diode array detector (DAD) with high sensitivity 60 mm flow cell and ESI-MSD iQ single quadrupole.

Buffer A	50 mM NH_4OAc, pH = 6.0
Buffer B	Acetonitrile
Flowrate	1 mL/min
Temperature	20 °C (column oven)
column	Poroshell 120 EC-C18 column<br>3 x 50 mm (2.7 µm)<br>Agilent P.N. 699975-302

Min	%A	%B
0	100	0
1	100	0
5	50	50
5.5	0	100
6.5	0	100
7	100	0
8	100	0

Every sample is followed by 2 min equilibration with 100 %A.

Synthesis of AMP analogues 2a-5a

General procedure

The respective adenosine derivative (1 eq.) was solved in 20 mL dry trimethyl phosphate (TMP) under an argon atmosphere. The solution was cooled to 0 °C and freshly distilled $POCl_3$ (1.6 eq.) was added dropwise. After stirring for 3 hours, the reaction was quenched with an aqueous solution of triethylammonium bicarbonate (TEAB, 1 M, 35 mL) until the gassing stopped and stirred for another 10 min. To remove residual TMP, the solution was washed with methyl *tert*-butyl ether (MTBE, 5 x 60 mL), ethyl acetate (1 x 60 mL) and MTBE (5 x 60 mL). Afterwards, residual ether was removed from the aqueous phase through argon bubbling for 15 min. Subsequently, the solution was concentrated under vacuum to a total volume of approximate 10 mL. The purification of each compound was performed by C18 flash chromatography on puriFlash XS520 (Interchim), with a C18-AQ HP Gold 100 g column (Teledyne ISCO).

Buffer A	50 mM NH_4OAc, pH = 6
Buffer B	Acetonitrile
Flowrate	60 mL/min
Temperature	room temperature
column	C18-AQ HP Gold 100 g

Min	%A	%B
0	100	0
4	100	0
16	50	50
17	0	100
20	0	100

For the following ion-exchange, 10 g of Dowex 50WX8 ion-exchange resin (Thermo Scientific) was washed with 40 mL ddH_2O and activated with 40 mL of NaOH (2 M) by stirring for 20 minutes. The suspension was poured into a 12 mL syringe containing a cotton plug. The product solution was added on top of the resin and rinsed through it. Fractions were collected by adding more ddH_2O. The presence of the product was monitored by thin-layer chromatography, and the fractions containing the desired AMP analogue were grouped and lyophilized.

Synthesis of 2-chloroadenosine-5′-monophosphate (2a)

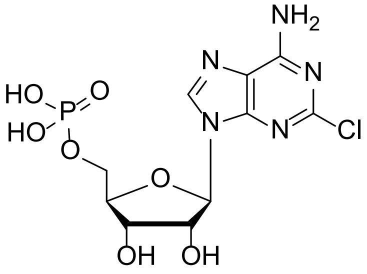

2-chloroadenosine-5′-monophosphate was synthesised according to the general procedure. 2-chloroadenosine (602 mg, 2.10 mmol) was used as starting material. The product was obtained as a white solid (796 mg, 1.98 mmol, 94 %).

FTMS (ESI-): Calculated mass for $C_{10}H_{12}ClN_5O_7P^-$ 380.0163 Da, found $[M-H]^-$ = 380.0167 *m/z*.

^{1}H NMR (500 MHz, D_2O) δ (ppm) = 4.03 (m, 2H), 4.35 (m, 1H), 4.50 (m, 1H), 4.72 (m, 1H), 5.98 (d, *J* = 5.3 Hz, 1H). 8.49 (s, 1H).

^{13}C NMR (126 MHz, D_2O) δ (ppm) = 155.69, 153.50, 149.68, 140.14, 117.15, 87.08, 84.47, 74.62, 70.44, 63.37.

^{31}P NMR (202 MHz, D_2O) δ (ppm) = 3.77 (s).

Synthesis of 2-fluoroadenosine-5′-monophosphate (3a)

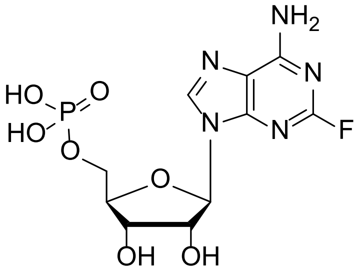

2-fluoroadenosine-5′-monophosphate was synthesised according to the general procedure. 2-fluoroadenosine (286 mg, 1.0 mmol) was used as starting material. The product was obtained as a white solid (263 mg, 0.68 mmol, 68 %).

FTMS (ESI-): Calculated mass for $C_{10}H_{12}FN_5O_7P^-$ 364.0458 Da, found $[M-H]^-$ = 364.0461 *m/z*.

^{1}H NMR (500 MHz, D_2O) δ (ppm) = 4.13 (m, 2H), 4.36 (m, 1H), 4.49 (m, 1H), 4.69 (m, 1H), 5.94 (d, *J* = 5.4 Hz, 1H), 8.34 (s, 1H).

^{13}C NMR (126 MHz, D_2O) δ (ppm) = 159.56, 157.87, 156.59, 150.06, 139.73, 116.45, 87.14, 83.90, 74.36, 70.27, 64.17.

^{31}P NMR (202 MHz, D_2O) δ (ppm) = 0.72 (s).

Synthesis of 2-aminoadenosine-5′-monophosphate (4a)

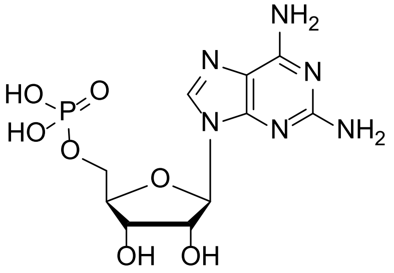

2-aminoadenosine-5′-monophosphate was synthesised according to the general procedure. 2-aminoadenosine (564 mg, 2.0 mmol) was used as starting material. The product was obtained as a white solid (502 mg, 1.3 mmol, 66 %).

FTMS (ESI+): Calculated mass for $C_{10}H_{14}N_6Na_2O_7P^+$ 407.0457 Da, found $[M+2Na-H]^+$ = 407.0450 *m/z*.

^{1}H NMR (500 MHz, D_2O) δ (ppm) = 4.03 (m, 2H), 4.31 (m, 1H), 4.47 (m, 1H), 4.70 (m, 1H), 5.87 (d, *J* = 5.7 Hz, 1H), 8.13 (s, 1H).

^{13}C NMR (126 MHz, D_2O) δ (ppm) = 159.16, 155.10, 150.66, 137.35, 112.50, 86.43, 83.99, 73.99, 70.47, 63.66.

^{31}P NMR (202 MHz, D_2O) δ (ppm) =2.96 (s).

Synthesis of N^6-methyladenosine-5′-monophosphate (5a)

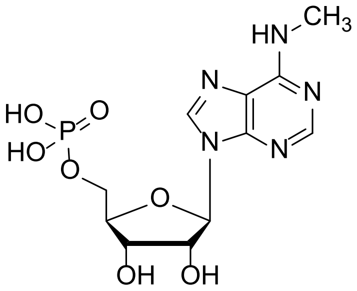

N^{6-}methyladenosine-5′-monophosphate was synthesised according to the general procedure. N^6-methyladenosine (259 mg, 0.92 mmol) was used as starting material. The product was obtained as a white solid (327 mg, 0.86 mmol, 93 %).

FTMS (ESI-): Calculated mass for $C_{11}H_{15}N_5O_7P^-$ 360.0709 Da, found $[M-H]^-$ = 360.0712 *m/z*.

^{1}H NMR (500 MHz, D_2O) δ (ppm) = 2.94 (s, 3H), 4.15 (m, 2H), 4.37 (m, 1H), 4.48 (m, 1H),4.68 (m, 1H), 6.00 (d, *J* = 5.6 Hz, 2H) 7.99 (s, 1H), 8.29 (s, 1H).

^{13}C NMR (126 MHz, D_2O) δ (ppm) = 154.06, 151.94, 146.97, 138.80, 118.25, 86.93, 83.76, 74.37, 70.25, 64.32, 27.19.

^{31}P NMR (202 MHz, D_2O) δ (ppm) = 0.38 (s).

Molecular Modelling

Structural models were generated with AlphaFold 2.3 in multimer mode running on a local high-performance computing cluster. The full tetrameric model of EbPPK2 was aligned to the reported tetrameric MrPPK2 structure (PDB 5LD1) using the MatchMaker tool in UCSF ChimeraX 1.7.[1] After alignment, the coordinates of the nucleotides were transferred from the MrPPK2 structure to the predicted EbPPK2 model. The composite structure was energy minimized with the YASARA force field minimization server[2] and the nucleotide-binding pocket was analysed in UCSF Chimera X 1.7.

EbPPK2 construct:

Cloning

pET28a(+)-EbPPK2-His_6 cloned with NcoI/XhoI restriction sites was ordered at BioCat (Heidelberg).

DNA sequence

5′ATGGCAAATATCTACAAGATCGATAAGCTGAATAACTTTAACCTGAATAACCATAAGACC
GATGATTATAGTCTGTGTAAAGATAAAGACACCGCCCTGGAACTGACCCAGAAAAATATTCA
GAAAATCTATGACTACCAGCAGAAACTGTATGCCGAAAAGAAAGAAGGTCTGATTATTGCAT
TTCAGGCAATGGATGCAGCCGGCAAAGATGGCACCATTCGCGAAGTGCTGAAAGCACTGGCC
CCGCAGGGCGTTCATGAAAAACCGTTTAAAAGTCCGAGCAGTACCGAACTGGCACATGATTA
TCTGTGGCGCGTTCATAATGCAGTGCCGGAAAAAGGTGAAATTACCATTTTTAATCGCAGTC
ATTACGAAGATGTGCTGATTGGTAAAGTTAAAGAACTGTATAAGTTCCAGAACAAAGCCGAT
CGTATTGATGAAAATACCGTTGTGGATAATCGTTATGAAGATATTCGTAATTTCGAGAAATA
CCTGTATAACAATAGCGTTCGCATTATTAAGATCTTTCTGAATGTTAGTAAGAAGGAACAGG
CAGAACGCTTTCTGAGTCGTATTGAAGAACCGGAAAAGAATTGGAAATTTTCAGATAGCGAT
TTCGAAGAACGTGTTTATTGGGATAAATATCAGCAGGCATTTGAAGATGCCATTAATGCAAC
CAGTACCAAAGATTGCCCGTGGTATGTTGTGCCGGCAGATCGCAAATGGTATATGCGTTATG
TTGTTAGCGAAATTGTGGTTAAAACCCTGGAAGAAATGAATCCGAAATATCCGACCGTTACC
AAAGAAACCCTGGAACGTTTTGAAGGTTATCGTACCAAACTGCTGGAAGAATATAATTATGA
TCTGGATACCATCCGTCCGATTGAAAAACTCGAGCACCACCACCACCACCACTGA 3′

Amino acid sequence

This construct (EbPPK2-His_6): MW: 36.566 kDa, Molecular extinction coefficient: 57425 $M^{-1} \cdot cm^{-1}$

```
MANIYKIDKLNNFNLNNHKTDDYSLCKDKDTALELTQKNIQKIYDYQQKLYAEKKEGLIIAF
QAMDAAGKDGTIREVLKALAPQGVHEKPFKSPSSTELAHDYLWRVHNAVPEKGEITIFNRSH
YEDVLIGKVKELYKFQNKADRIDENTVVDNRYEDIRNFEKYLYNNSVRIIKIFLNVSKKEQA
ERFLSRIEEPEKNWKFSDSDFEERVYWDKYQQAFEDAINATSTKDCPWYVVPADRKWYMRYV
VSEIVVKTLEEMNPKYPTVTKETLERFEGYRTKLLEEYNYDLDTIRPIEKLEHHHHHH
```

EbPPK2 expression and purification

For EbPPK2 production, *E. coli* BL21(DE3) cells were transformed with pET28a(+)-EbPPK2-His_6 by electroporation, regenerated in SOC media and cultivated in LB medium with kanamycin at 37 °C and 180 rpm for 16 h. 50 mL of this overnight culture were added to 1 L of LB medium with kanamycin and grown to an OD_{600} of 0.8. Expression was induced with 0.5 mM IPTG at 20 °C for 16 hours. Cells were harvested by centrifugation (5000 xg, 4 °C, 30 min) and pellets were stored at -80 °C.

Pellets were resuspended in Buffer A (50 mM Tris-HCl, pH 7.5, 300 mM NaCl, 10 mM imidazole, 10 % glycerol) and lysed by sonication (30 % amplitude, 0.5 second on/off pulses for 3 min, 3 times) by a Sonopuls GM3100 (Bandelin). Cell debris was removed by centrifugation (11000 xg, 4 °C, 30 min), and the supernatant was filtered through a 0.2 µm syringe filter and loaded into a Superloop™. Protein purification was performed by IMAC using a HisTrap FF 5 mL (GE Healthcare) column connected to a ÄKTA Purifier system (GE Healthcare). The protein was eluted with Buffer B (50 mM Tris pH 7.5, 300 mM NaCl, 500 mM imidazole, 10 % glycerol). Fractions containing EbPPK2 were concentrated and the buffer was exchanged to Buffer C (50 mM Tris pH 7.5, 300 mM NaCl, 10 % glycerol) using Amicon Ultra-15 centrifuge filters (UFC903024, 30 kDa cut off). The concentration was determined by BSA calibration or absorbance at 280 nm. Aliquots were flash frozen in liquid nitrogen and stored at -80 °C.

EbPPK2 characterization

Gel filtration Chromatography

0.16 mg of purified EbPPK2 protein (in 25 mM HEPES pH 7.4, 300 mM NaCl, 1 mM MgCl2, 1 mM TCEP) were loaded on an EnRich sec650 size exclusion column (BioRad), previously equilibrated in PBS (137 mM NaCl, 2.7 mM KCl, 10 mM Na_2HPO_4, 1.8 mM KH_2PO_4). The sample was eluted at a flow rate of 0.4 mL · min^{-1}. To obtain the molecular mass, the gel filtration standard #1511901 (BioRad) was applied under the same conditions. The EbPPK2 sample elutes as monochromatic peak at 12.27 mL corresponding to 144 kDa.

Thermal shift assay

The melting temperature of EbPPK2 in different buffers and ionic strength was determined using the RUBIC Buffer Screen (Molecular Dimensions), following the manufacturer´s protocol. In short, 21 µL of each buffer of the RUBIC Buffer Screen were transferred to a PCR-microplate. On ice, 2 µL of a 40 µM solution of EbPPK2 (in 25 mM Tris-HCl pH 7.5, 150 mM NaCl) were added to each well. 2 µL of a freshly prepared SYPRO Orange solution (Invitrogen, S6651, pre-diluted to 62X) were dispensed into each well, and the PCR-microplate was sealed with a clear adhesive tape. The microplate was placed in a RT-PCR machine pre-equilibrated at 10 °C. A temperature gradient from 10 °C to 95 °C with a ramp rate of 1 °C/min was used. Data collection was performed at a wavelength of λ = 610 nm. Melting points were calculated using the 1st derivative of the melting curves.[3]

EbPPK2 analytical scale reactions

For analytical reactions, 1 mM AMP or analogues (**1a**-**10a**) were incubated in 20 mM Tris (pH 8) with 20 mM $MgCl_2$, 6.1 g/L polyphosphate and 4 µM EbPPK2 in a total volume of 20 µL at 30 °C. Samples were taken after 2 min and 60 min, the enzyme was denatured at 85 °C for 2 min, and the samples were centrifuged (15 minutes, 21000x g, 4 °C) to remove precipitated protein. The supernatant (1 µL) was analysed *via* LC-DAD-Q-MS.

EbPPK2 preparative scale reactions

For preparative reactions, EbPPK2 (4 µM) in 20 mM Tris (pH 8) with 20 mM $MgCl_2$ and 6.1 g/L polyphosphate was preincubated at 30 °C, and the reactions were started by the addition of AMP or analogues to 5 mM (**1a**-**5a**). Reactions were run in a total volume of 2 mL for 2 min at 30 °C. The enzyme was then denatured at 85 °C for 2 min, and the reactions were centrifuged (15 minutes, 21000x g, 4 °C) to remove precipitated protein.

Purification of ATP analogues

For the purification of the ATP analogues (**1c**-**5c**), anion-exchange chromatography was performed on a ÄKTA Purifier system (GE Healthcare) using a HiPrep Q FF 16/10 column (Cytiva GE28936543). The system was equilibrated in ddH_2O with a flow rate of 5.0 mL/min. A preparative EbPPK2 reaction (2 mL) was mixed with 8 mL ddH_2O and loaded into a 10 mL Superloop^{TM} for injection.

Buffer A	ddH_2O
Buffer B	100 mM $NaClO_4$ (pH = 4.2)
Flowrate	5 mL/min
Temperature	room temperature
column	HiPrep Q FF 16/10

Min	%A	%B
0	100	0
6	100	0
6	55	45
10	55	45
30	0	100
40	0	100

The absorbance at λ = 254 nm and the conductivity were continuously monitored. After each purification, the column was washed with 30 mL of 500 mM $NaClO_4$ (pH = 4.2) and equilibrated with 40 mL ddH_2O.

Fractions containing the desired ATP analogue (**1c**-**5c**) were pooled and the volume of the solution was reduced to 1-2 mL through lyophilization. Note that drying of the ATP analogue solution to completion will lead to hydrolysis of the ATP derivative to the corresponding AMP derivative. To precipitate the ATP analogues and remove residual $NaClO_4$, the ATP solution was mixed with 50 volumes ice cold acetone and then

incubated at -20 °C for 2 hours. After centrifugation (30 minutes, 3220x g, 4 °C) the supernatant was decanted, and the solid was washed with acetone (cooled to -20 °C). After centrifugation (10 minutes, 3220x g, 4 °C) and decanting of supernatant, the product was dried at room temperature. Finally, each ATP analogue was dissolved in ddH_2O, and the concentration was determined by absorbance at 260 nm (280 nm for **4c**).

Polyadenylation of RNA

The purified ATP derivatives were used in polyadenylation reactions with yeast Poly(A) Polymerase (ScPAP, ThermoFisher Scientific) and a 30-nt RNA (Biolegio, sequence: 5′-GUGACCGCGGAUCGACUUCACCGCGCAGUG-3′). Poly(A) reactions were performed following the manufacturer's protocol. In short, 25 µL reactions were assembled with 0.2 µM RNA, 0.5 mM ATP or analogue, and 600 U ScPAP in 20 mM Tris-HCl pH 7, 0.6 mM $MnCl_2$, 0.02 mM EDTA, 0.2 mM DTT, 100 µg/mL acetylated BSA and 10% glycerol. Reactions were incubated 20 min at 37 °C, then 10 min at 65 °C to inactivate ScPAP. 5 µL aliquots were mixed 1:1 with 2x RNA loading dye (0.025 % SDS, 0.025 % xylene cyanol, 0.025 % bromophenol blue, and 0.5 mM EDTA in deionized formamide), denatured for 4 min at 95 °C, and resolved on denaturing urea-polyacrylamide gels (7.5 % or 10 %). Gels were run ~1 h at 25 or 30 W, stained using SYBR Gold (Invitrogen), and visualized using an Amersham Typhoon Biomolecular Imager.

Supplementary figures

Alphafold Structure Predictions

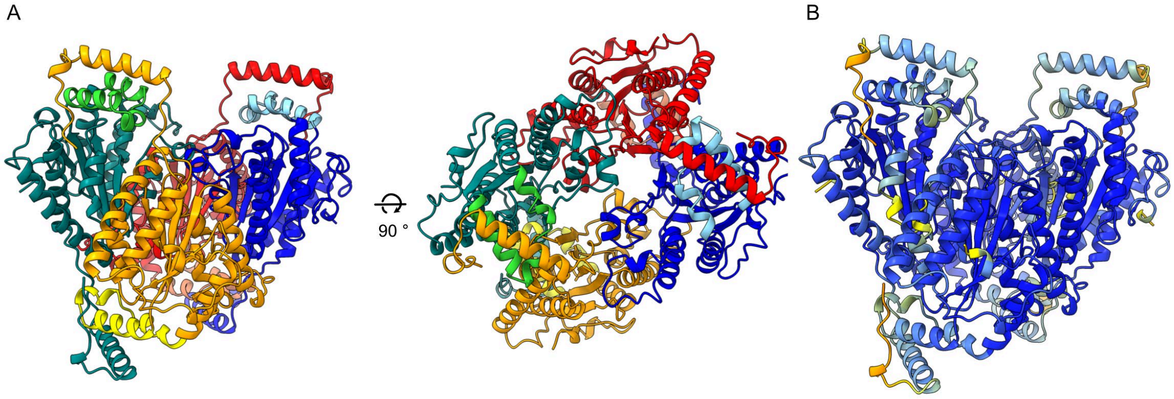

Figure S1: AlphaFold2-generated homotetrameric model of EbPPK2-III A) coloured by chain. The lid helices of the nucleotide binding pocket (termed helix 8a and 8b in MrPPK2) are coloured in brighter shades. B) AlphaFold2 model coloured by pLDDT score according to Jumper et al.[4] from high confidence (dark blue) to medium confidence (orange).

Preparative HPLC purification of 2a-5a

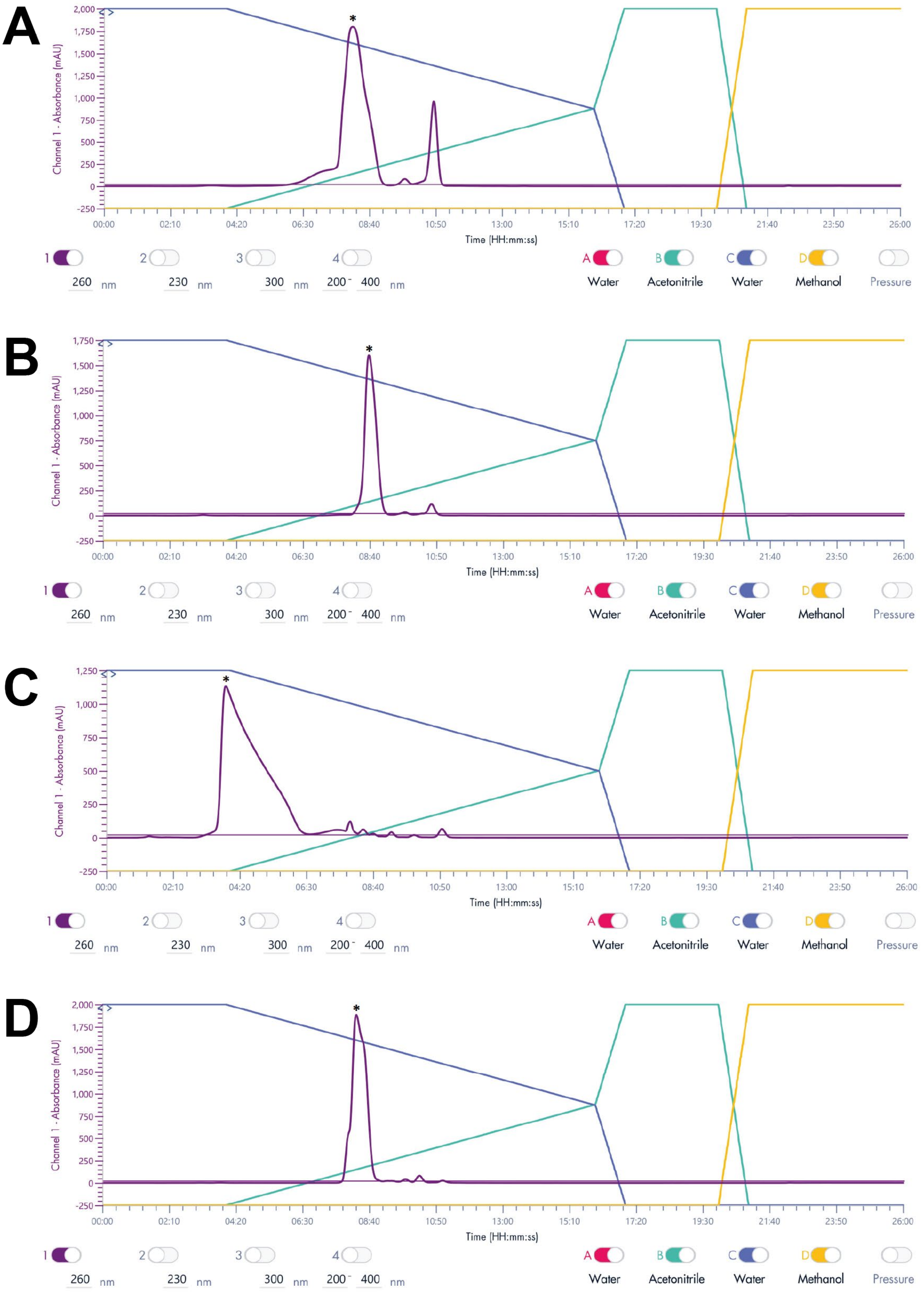

Figure S2: Purification of AMP analogues. A)-D) UV-chromatograms of the preparative C18-flash purification of **2a-5a**. Purification was performed on puriFlash XS520 (Interchim) using a C18-AQ HP Gold 100 g column (Teledyne ISCO). The fractions corresponding to the peaks marked with * were pooled.

FTMS spectra of 2a-5a

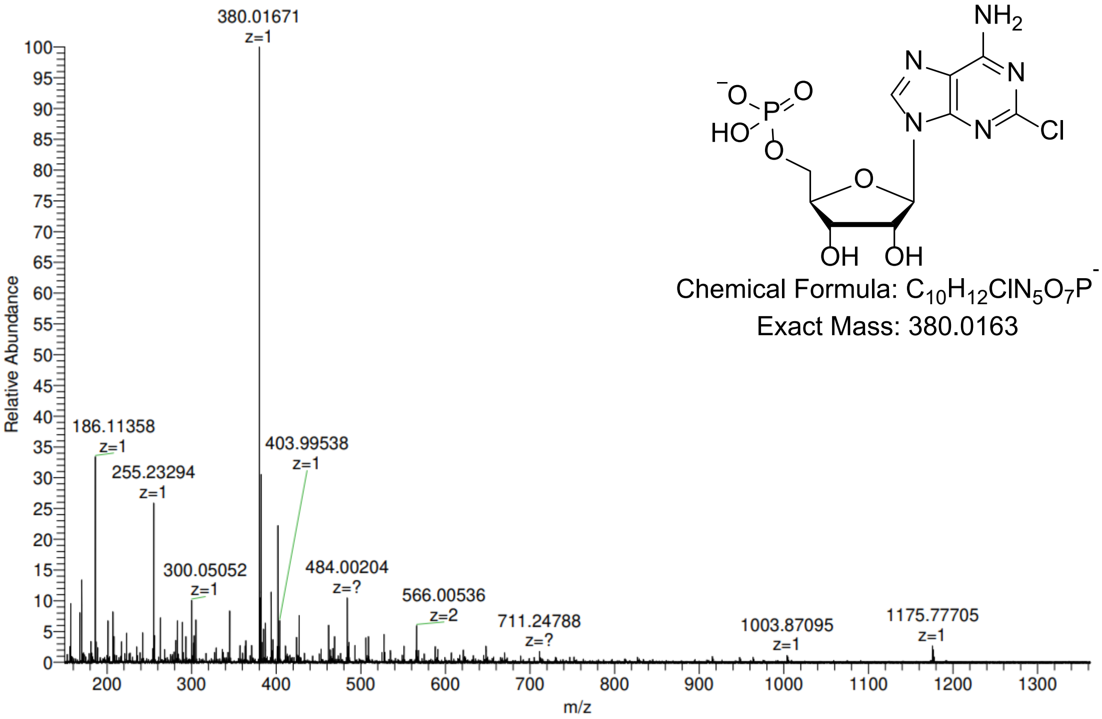

Figure S3: FTMS spectrum (ESI-) of **2a**. Calculated mass for $C_{10}H_{12}ClN_5O_7P^-$ 380.0163 Da, found $[M\text{-}H]^-$ = 380.0167 *m/z*.

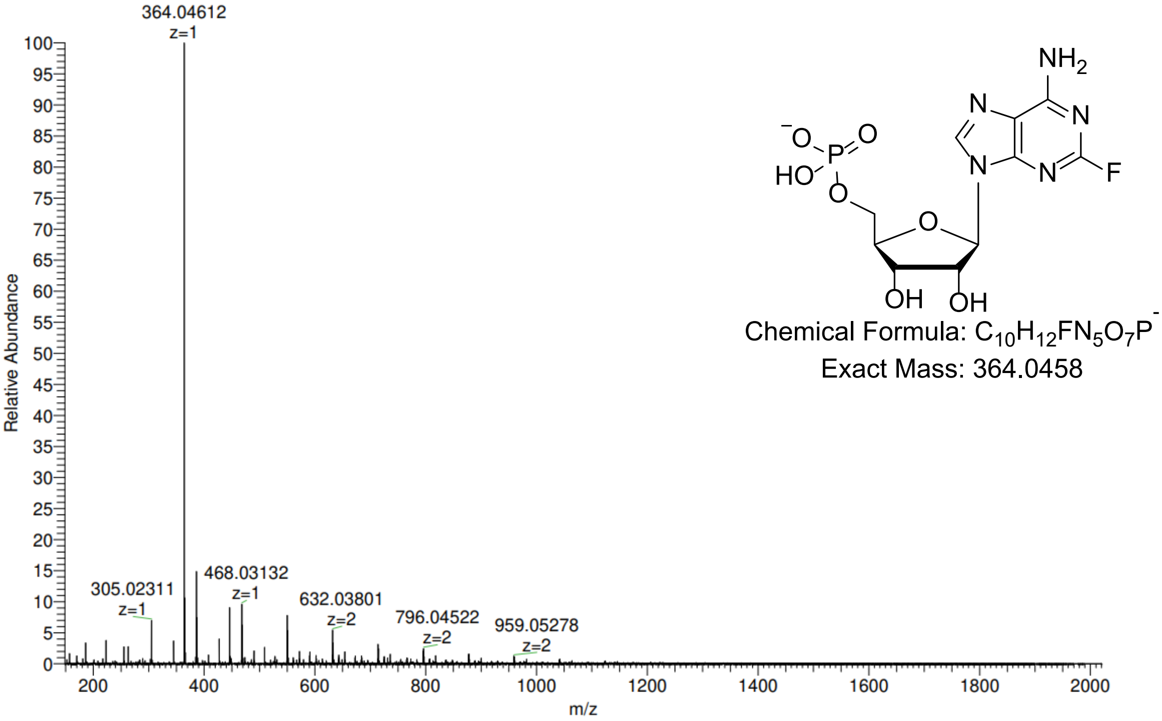

Figure S4: FTMS spectrum (ESI-) of **3a**. Calculated mass for $C_{10}H_{12}FN_5O_7P^-$ 364.0458 Da, found $[M\text{-}H]^-$ = 364.0461 *m/z*.

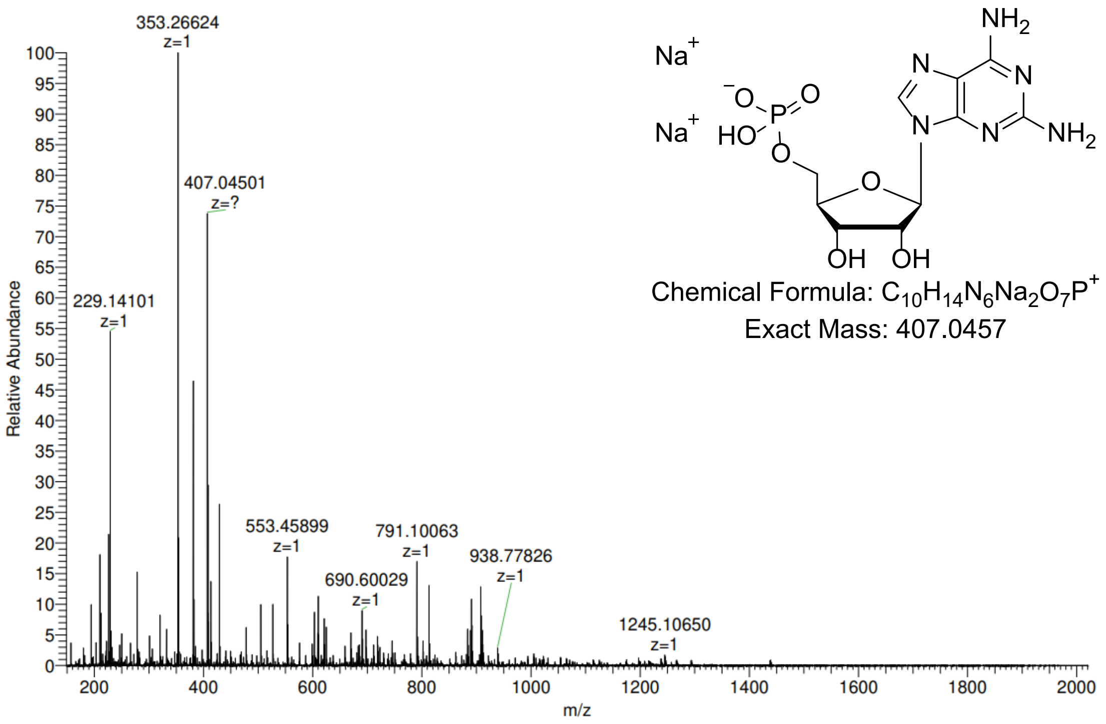

Figure S5: FTMS spectrum (ESI+) of **4a**. Calculated mass for $C_{10}H_{14}N_6Na_2O_7P^+$ 407.0457 Da, found $[M+2Na-H]^+$ = 407.0450 *m/z*.

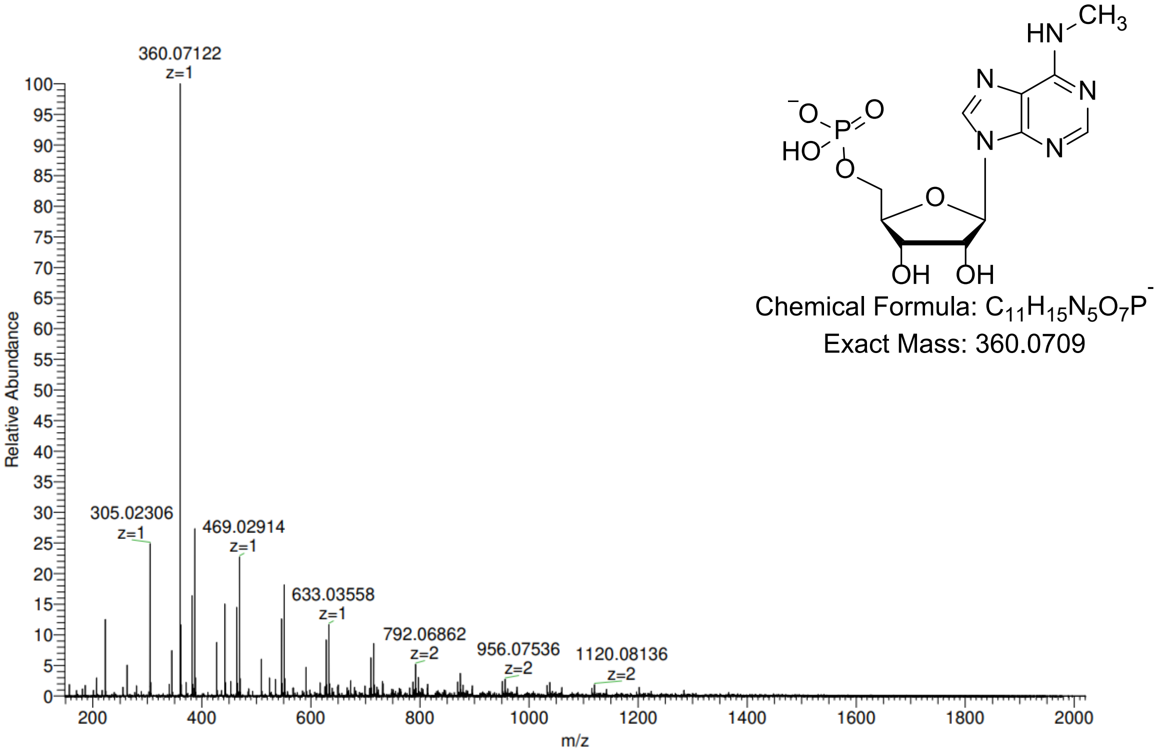

Figure S6: FTMS spectrum (ESI-) of **5a**. Calculated mass for $C_{11}H_{15}N_5O_7P^-$ 360.0709 Da, found $[M-H]^-$ = 360.0712 *m/z*.

NMR spectra of AMP analogues (2a-5a)

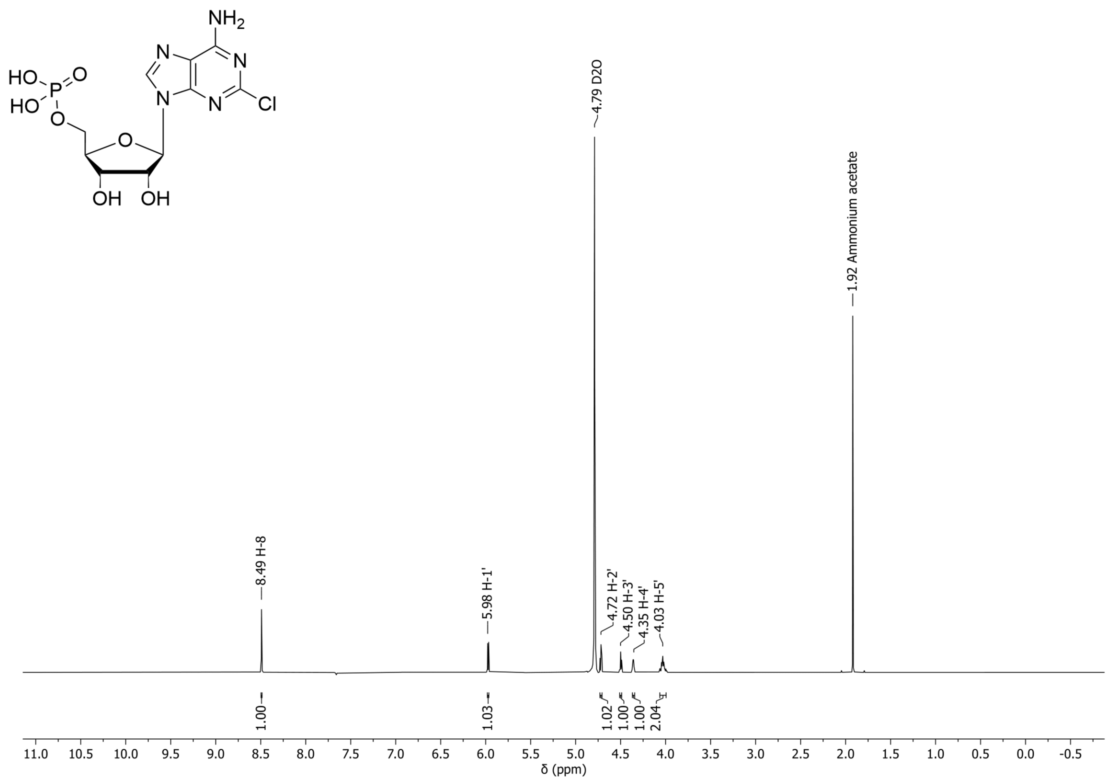

Figure S7: ^{1}H NMR spectrum of **2a**.

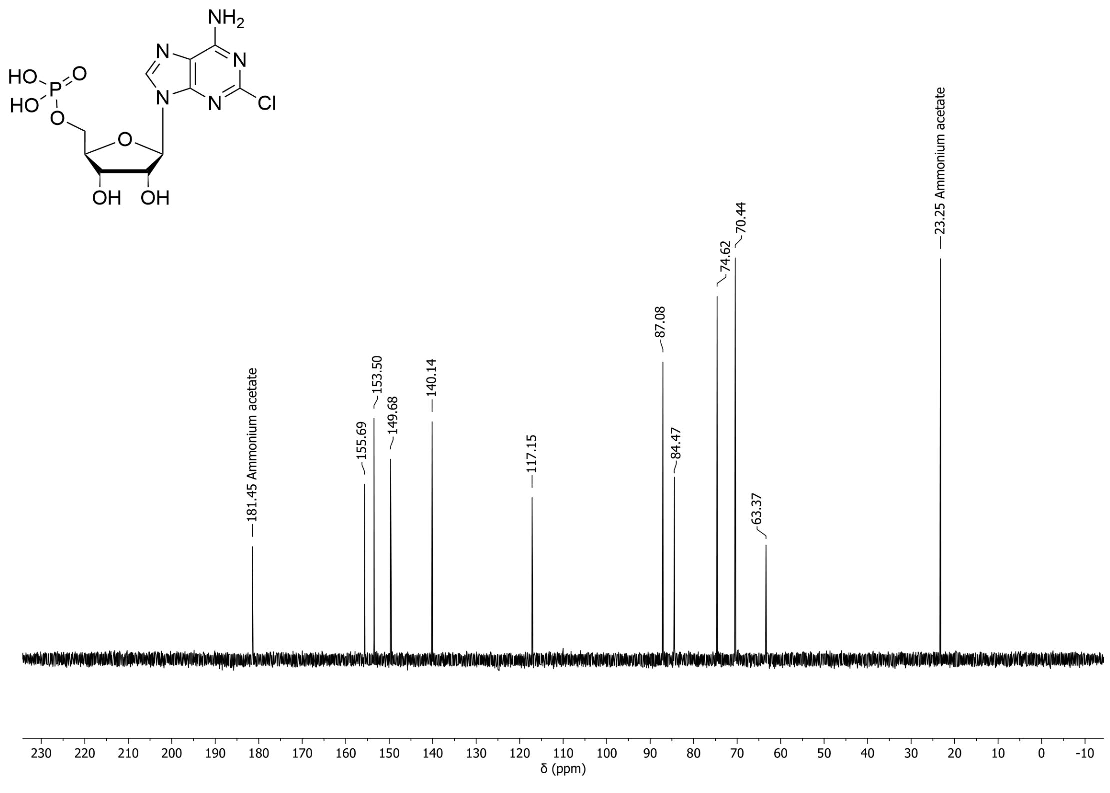

Figure S8: ^{13}C NMR spectrum of **2a**.

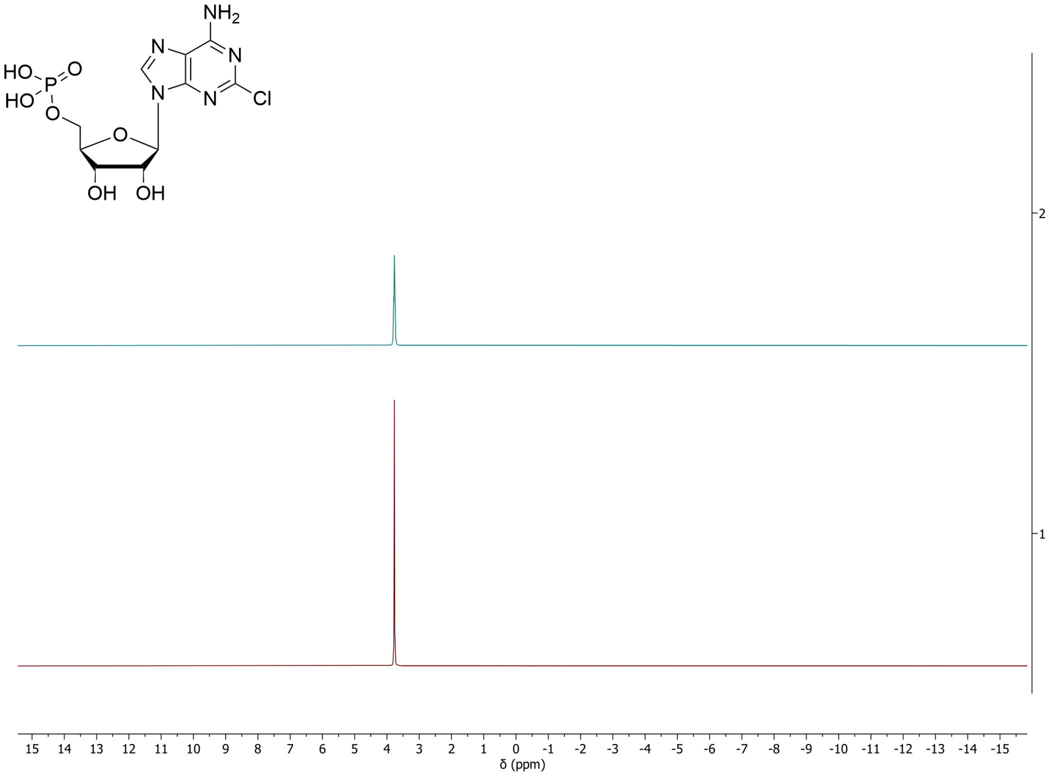

Figure S9: ^{31}P NMR spectrum of **2a**.

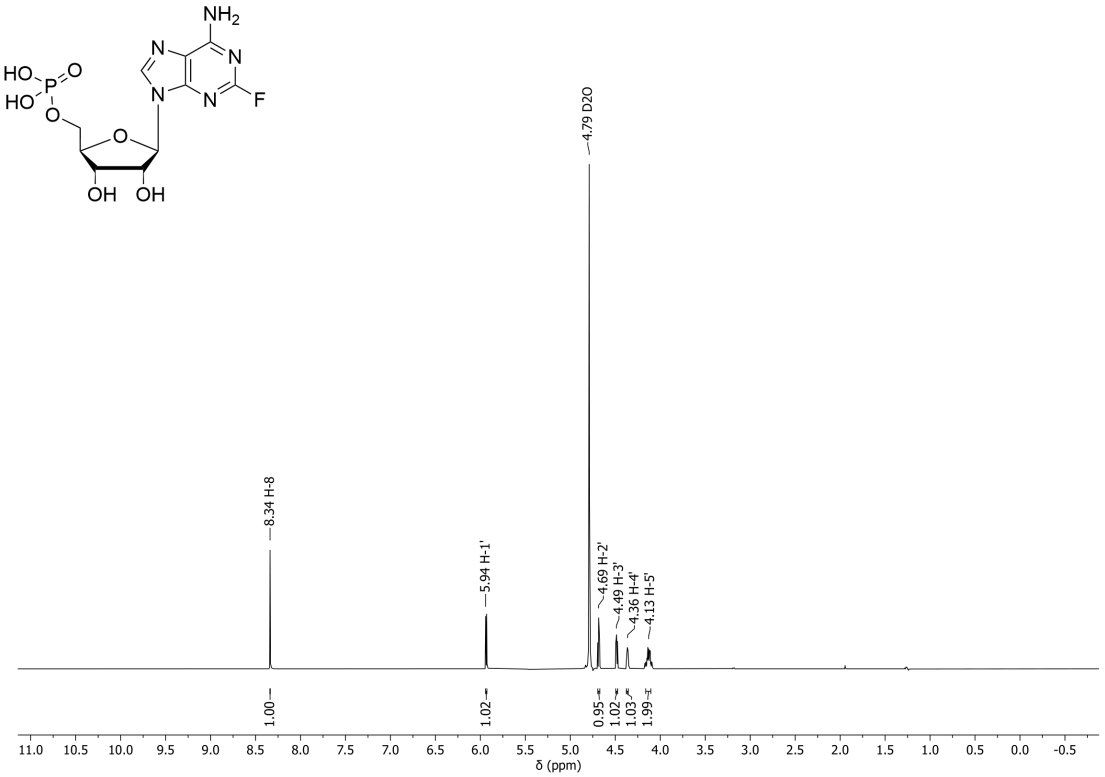

Figure S10: ^{1}H NMR spectrum of **3a**.

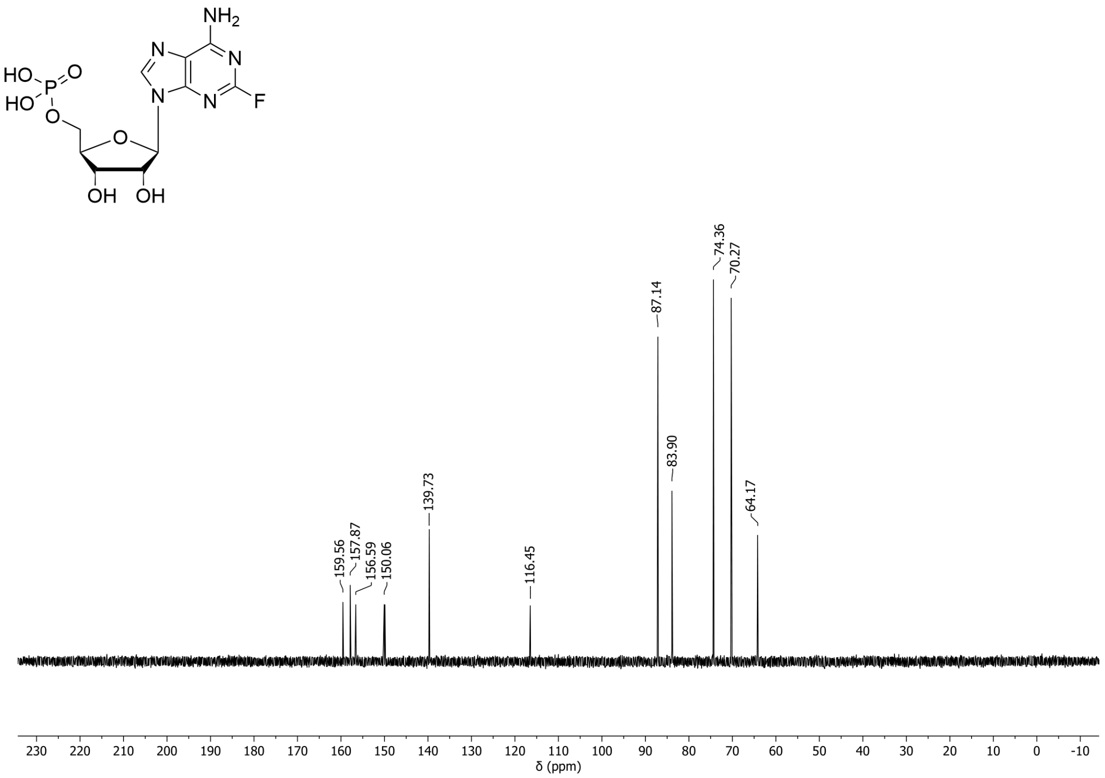

Figure S11: ^{13}C NMR spectrum of **3a**.

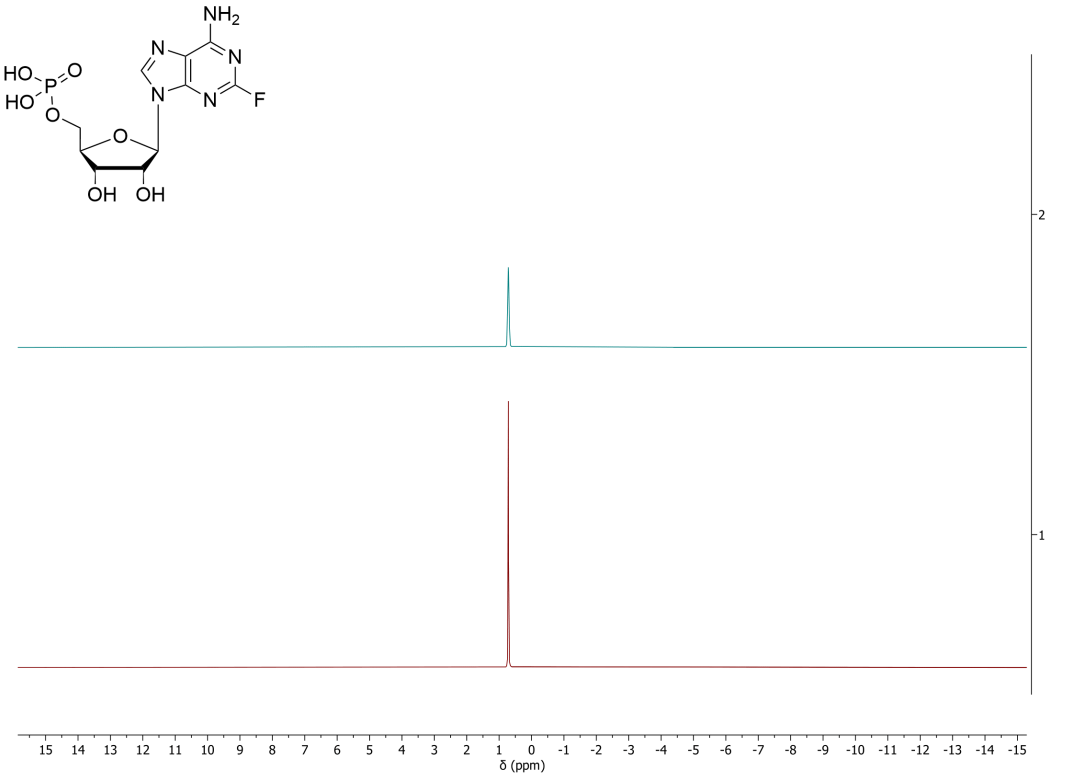

Figure S12: ^{31}P NMR spectrum of **3a**.

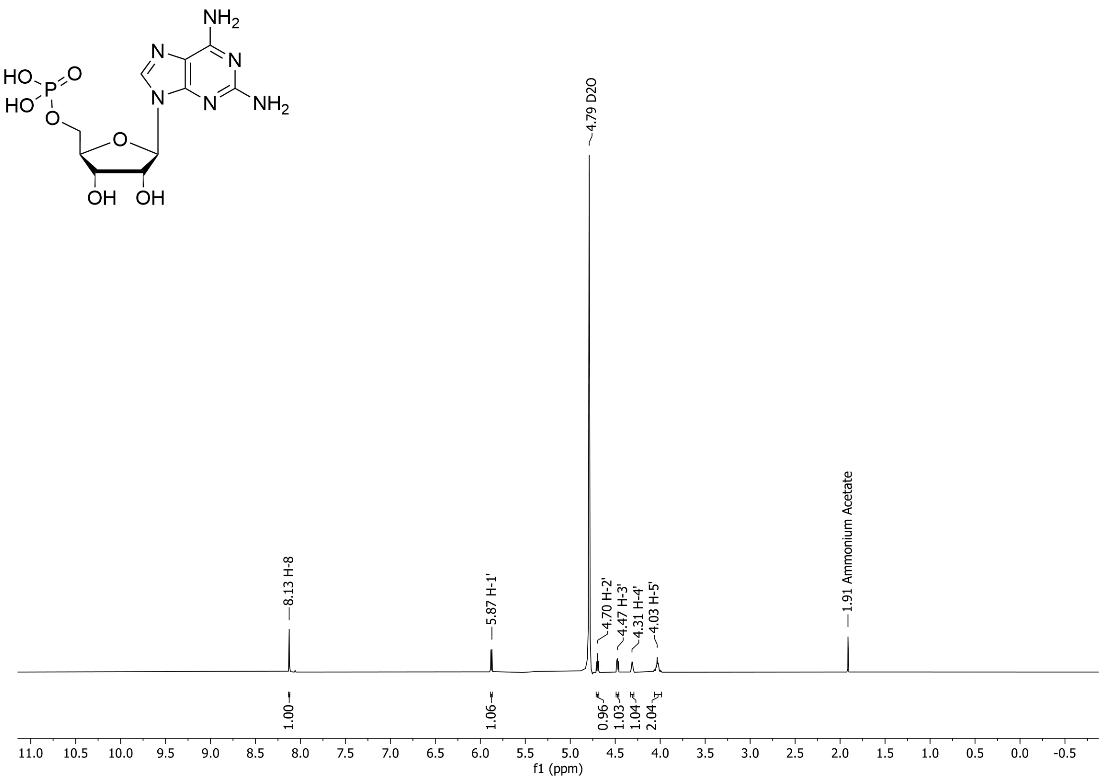

Figure S13: ^{1}H NMR spectrum of **4a**.

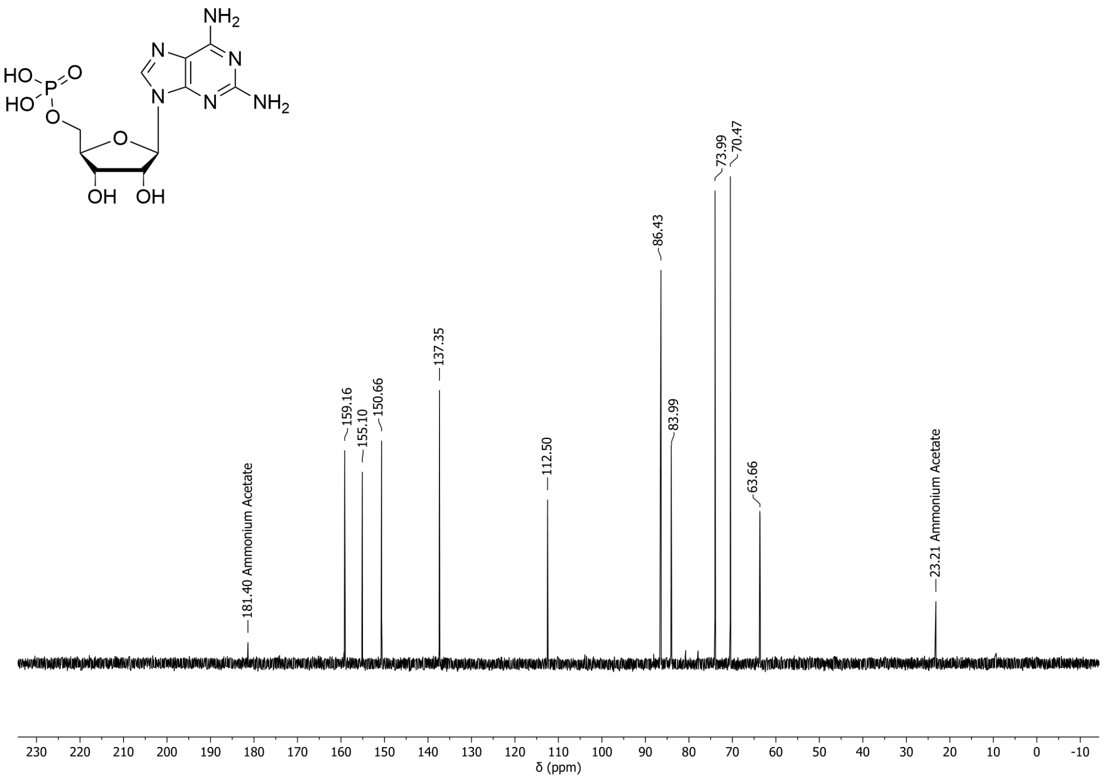

Figure S14: ^{13}C NMR spectrum of **4a**.

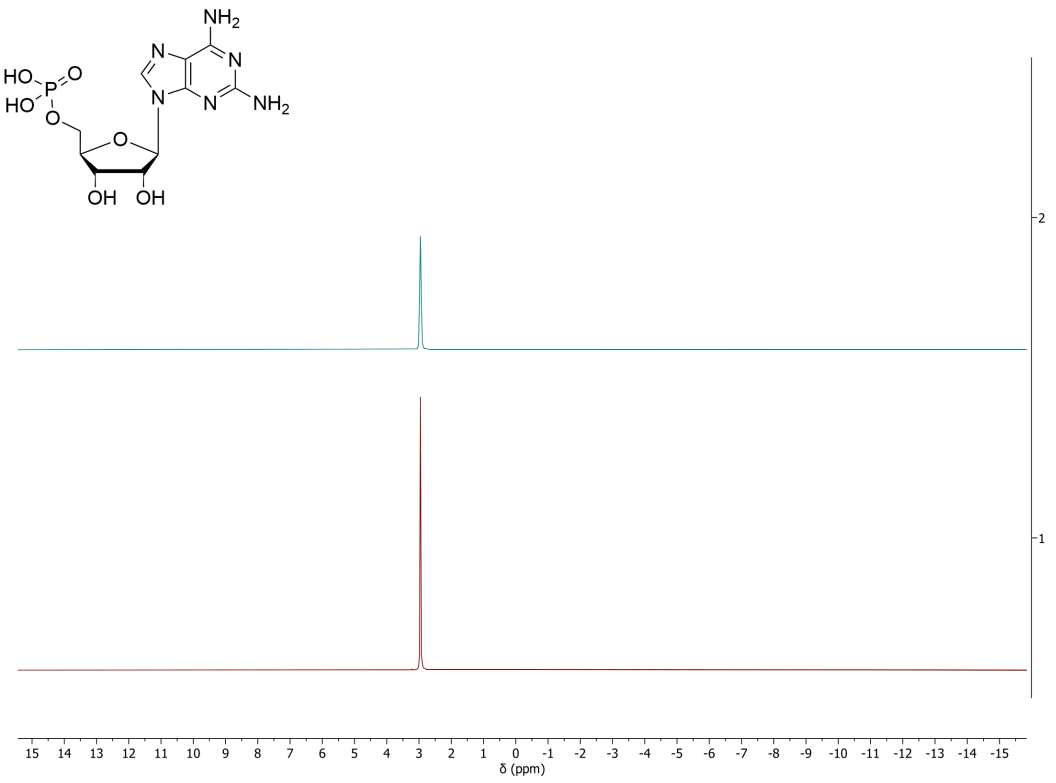

Figure S15: ^{31}P NMR spectrum of **4a**.

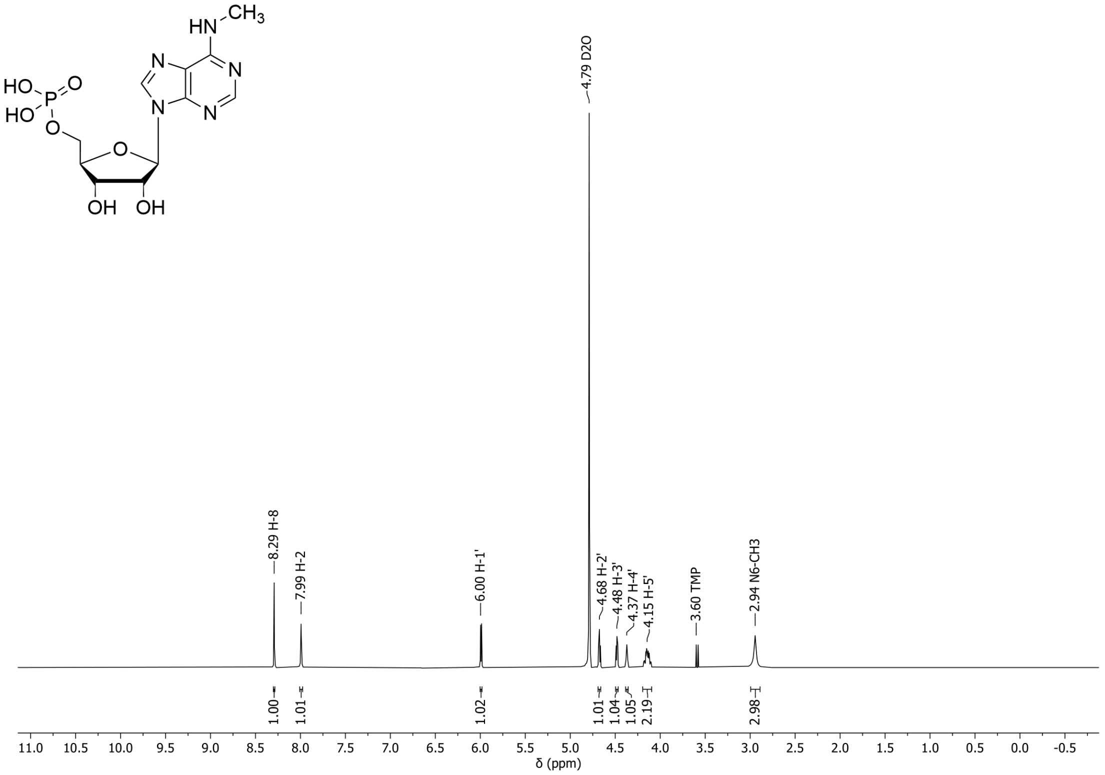

Figure S16: ^{1}H NMR spectrum of **5a**.

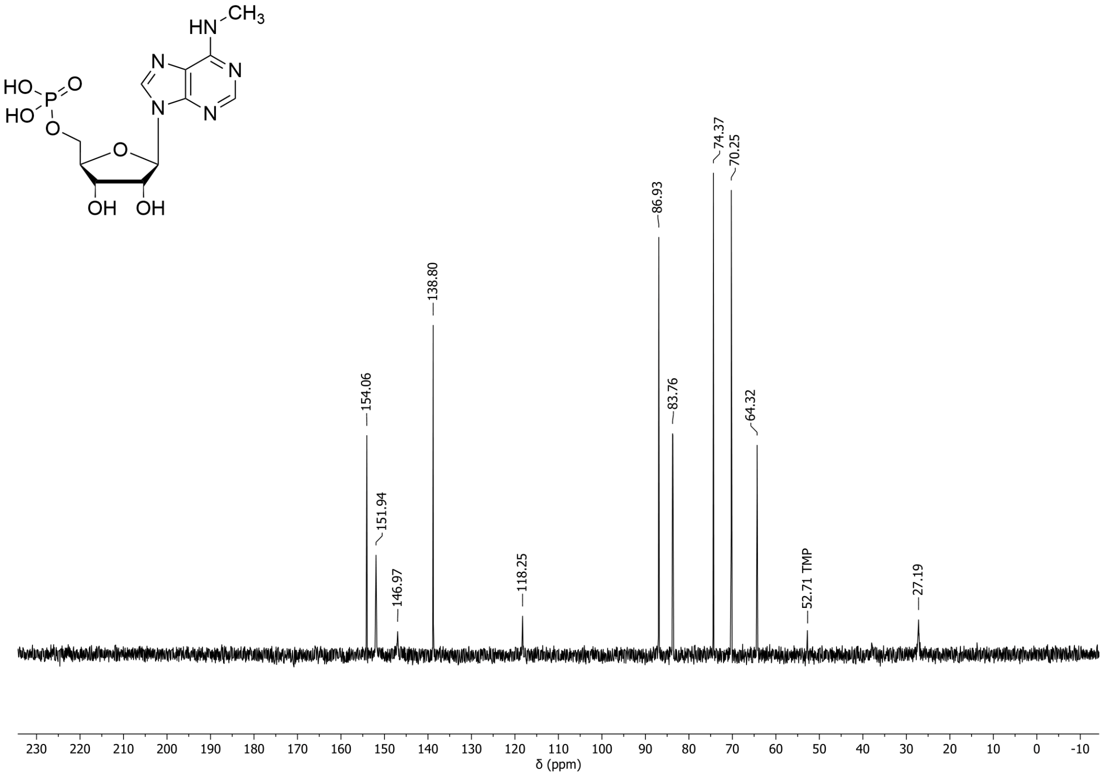

Figure S17: ^{13}C NMR spectrum of **5a**.

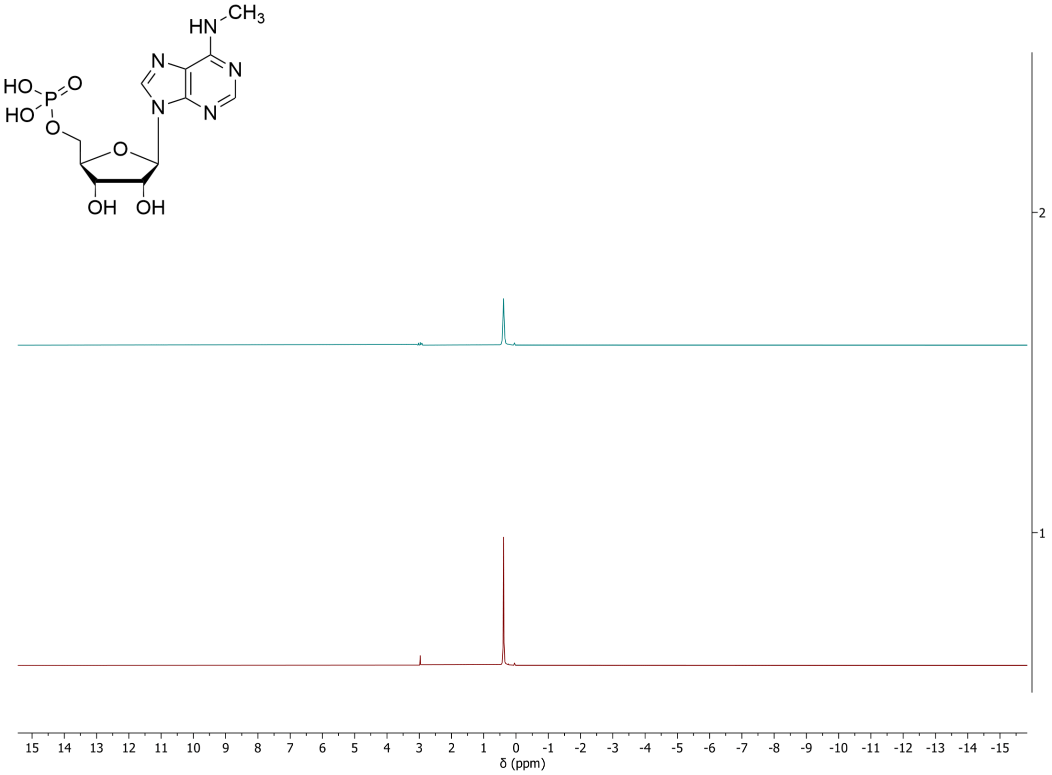

Figure S18: ^{31}P NMR spectrum of **5a**. Note that the peak at a chemical shift of δ = 2.97 ppm corresponds to residual traces of trimethyl phosphate.

Purification of EbPPK2

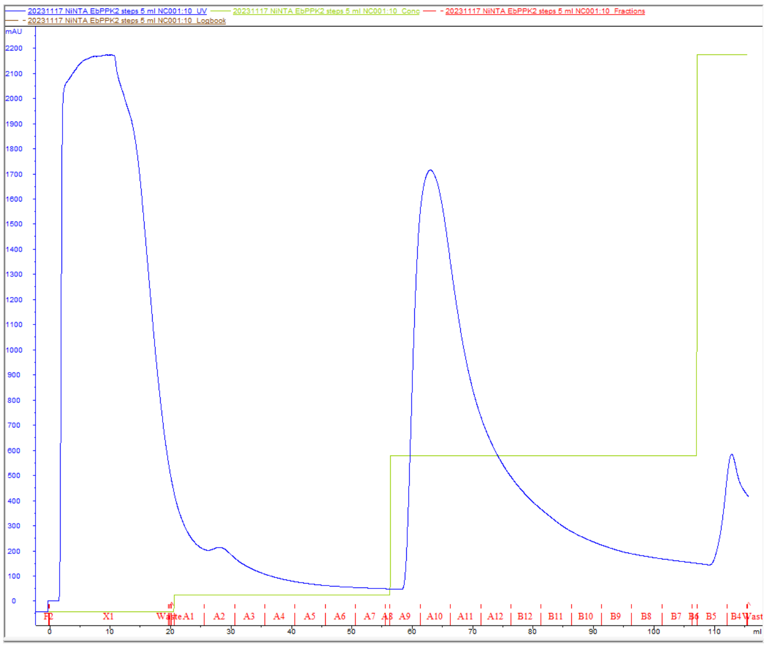

Figure S19: UV-chromatogram at 280 nm of the purification of EbPPK2 using a ÄKTA purifier equipped with a HisTrap FF (5 mL, GE Healthcare) column.

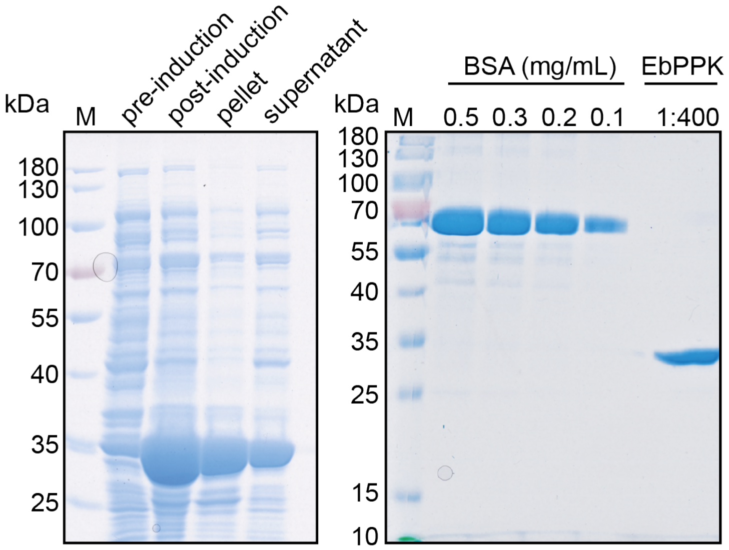

Figure S20: SDS-PAGE gel of EbPPK2 expression and purification. A) Electrophoretic separation of proteins from the pET28(a)+ EbPPK2-transformed BL21(DE3) cells. Gel shows cell suspension before (**pre-induction**) and after (**post-induction**) the induction of the protein production using IPTG, as well as insoluble (**pellet**) and soluble (**supernatant**) cell fractions after sonication and centrifugation. B) Different concentrations of Bovine serum albumin (BSA) and dilution of the purified EbPPK2. **M**: PageRuler Prestained Protein Ladder (Thermo Fisher Scientific). Gels (12 % polyacrylamide) were stained with Coomassie Blue G-250.

Multimer determination by gel filtration

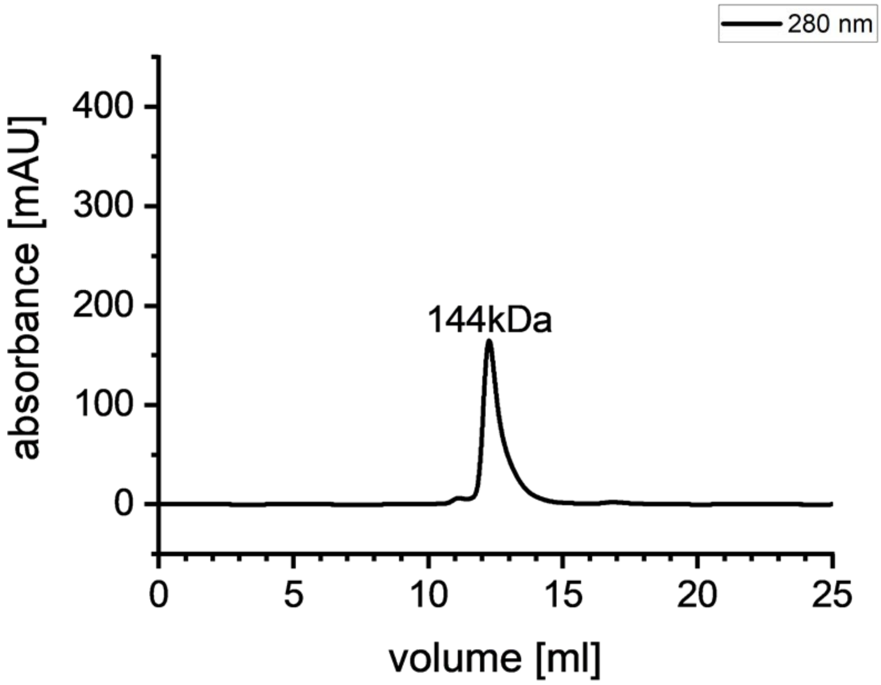

Figure S21: UV trace of EbPPK2 on an EnRich sec 650 10/300 column. Elution volume was compared to the gel filtration standard (BioRadCatalog # 1511901) and calculated to the indicated mass.

Thermal shift assay

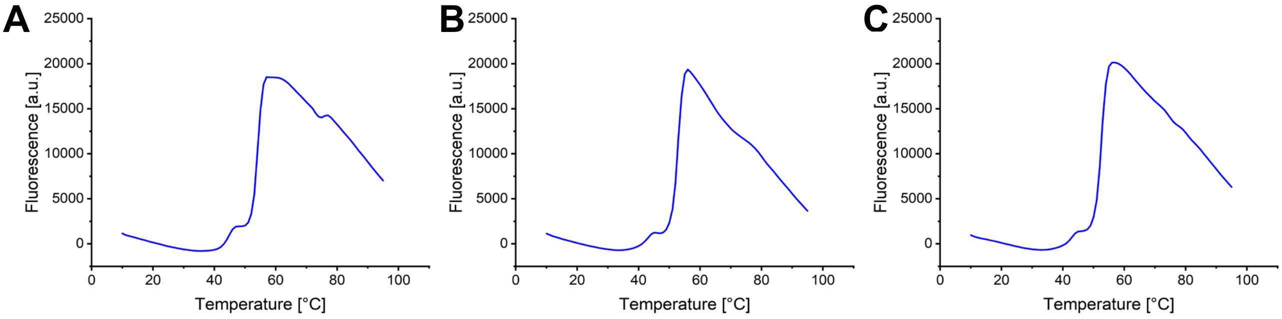

Figure S22: Melting curves of EbPPK2 using the RUBIC buffer screen. The three curves with the highest T_m values are shown. A)-C) refer to the buffer conditions F8, A10, and C10 respectively of the RUBIC Buffer screen. Each of these buffers contains 100mM phosphate, suggesting that phosphate ions have a stabilizing effect on EbPPK.

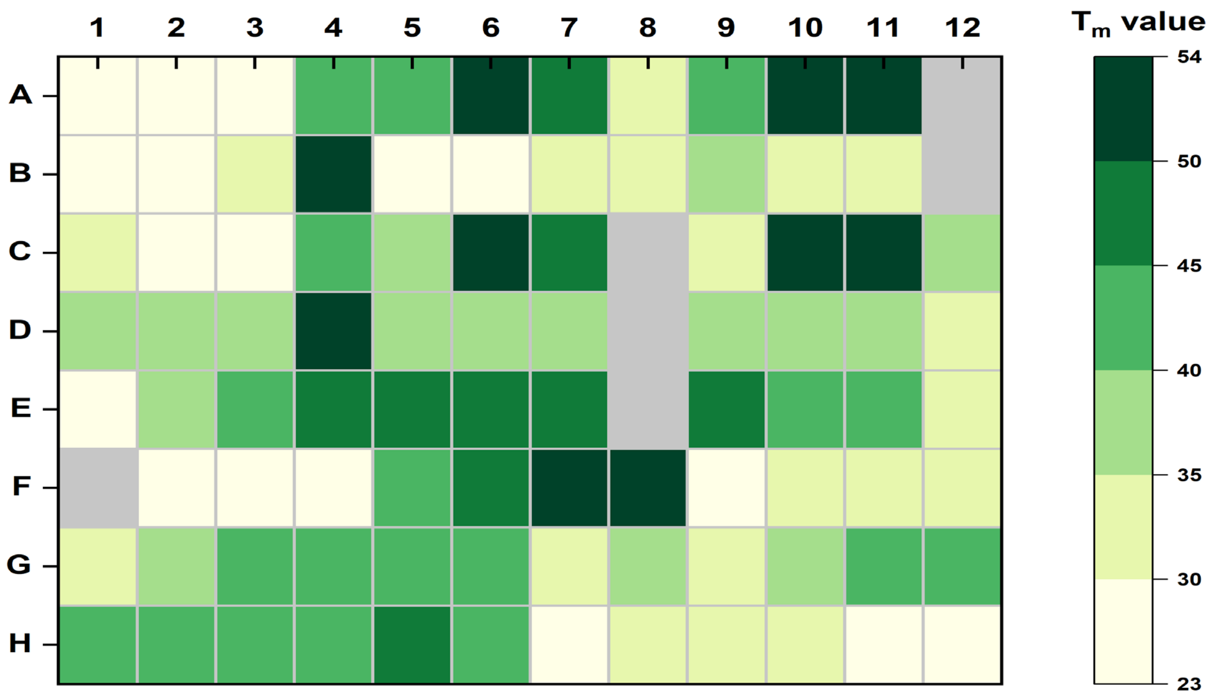

	1	2	3	4	5	6	7	8	9	10	11	12
**A**		100mM Citrate pH4	100mM Na acetate pH4.5	100mM Citrate, pH5	100mM MES pH6	100mM KPi pH6	100mM Citrate pH6	100mM Bis-Tris pH6.5	100 mM MES pH6.5	100mM NaPi pH7	100mM KPi pH7	100mM HEPES pH7
**B**	100mM MOPS pH7	100mM Ammon. acetate pH7.3	100mM Tris-HCl pH7.5	100mM NaPi 7.5	100mM Imidazol e pH7.5	100mM HEPES pH8	100mM Tris-HCl pH8	100mM Tricine pH8	100mM BICINE pH8	100mM BICINE pH8.5	100mM Tris-HCl pH8.5	100mM CHES pH9
**C**	250mM NaCl	100mM Citrate pH4 250 mM NaCl	100mM Na acetate pH4,5 250 mM NaCl	100mM Citrate pH5 250 mM NaCl	100mM MES pH6 250 mM NaCl	100mM KPi pH6 250 mM NaCl	100mM Citrate pH6 250 mM NaCl	100mM Bis-Tris pH6.5 250 mM NaCl	100mM MES pH6.5 250 mM NaCl	100mM NaPi pH7 250 mM NaCl	100mM KPi pH7 250 mM NaCl	100mM CHES pH7 250mM NaCl
**D**	100mM MOPS pH7 250 mM NaCl	100mM Ammon. acetate pH7.3 250 mM NaCl	100mM Tris-HCl pH7.5 250mM NaCl	100mM NaPi pH7.5 250 mM NaCl	100mM Imidazol e pH8 250 mM NaCl	100mM HEPES pH8 250mM NaCl	100mM Tris-HCl pH8 250 mM NaCl	100mM Tricine pH8 250 mM NaCl	100mM BICINE pH8 250 mM NaCl	100mM BICINE pH8.5 250 mM NaCl	100mM Tris-HCl pH8.5 250 mM NaCl	100mM CHES pH9 250 mM NaCl
**E**	100mM SPG pH4	100mM SPG pH4.5	100mM SPG pH5	100mM SPG pH5.5	100mM SPG pH6	100mM SPG pH6.5	100mM SPG pH7	100mM SPG pH7.5	100mM SPG pH8	100mM SPG pH8.5	100mM SPG pH9	100mM SPG pH10
**F**	20mM HEPES pH7.5	50mM HEPES pH7.5	125mM HEPES pH7.5	250mM HEPES pH7.5	20mM NaPi pH7.5	50mM NaPi pH7.5	125mM NaPi pH7.5	250mM NaPi pH7.5	20mM Tris-HCl pH8	50mM Tris-HCl pH8	125mM Tris-HCl pH8	250mM Tris-HCl pH8
**G**	50mM HEPES pH7.5 50 mM NaCl	50mM HEPES pH7.5 125 mM NaCl	50mM HEPES pH7.5 250 mM NaCl	50mM HEPES pH7.5 500 mM NaCl	50mM HEPES pH7.5 750 mM NaCl	50mM HEPES pH7.5 1000 mM NaCl	50mM Tris-HCl pH8 50 mM NaCl	50mM Tris-HCl pH8 125 mM NaCl	50mM Tris-HCl pH8 250 mM NaCl	50mM Tris-HCl pH8 500 mM NaCl	50mM Tris-HCl pH8 750 mM NaCl	50mM Tris-HCl pH8 1000 mM NaCl
**H**	50mM MES/Bis-Tris pH 6	50mM MES/Imidazole pH 6.5	50mM Bis-Tris/PIPES pH 6.5	50mM MOPS/Bis-Tris propane pH 7	50mM Phosphate/Citrate pH 7.5	50mM MOPS/Na HEPES pH 7.5	50mM BICINE/Tris pH 8.5	50mM Imidazole pH 7.5 100 mM NaCl	125mM Imidazole pH 7.5 100 mM NaCl	250mM Imidazole pH 7.5 100 mM NaCl	350mM Imidazole pH 7.5 100 mM NaCl	500mM Imidazole pH 7.5 100 mM NaCl

Fig S23. Full Rubic Buffer screen of EbPPK. A) Heatmap of melting temperatures (T_m), calculated using the first derivative of melting curves such as shown in Figure S20 above. Melting temperatures could not be determined for cells in grey. B) Buffer/ionic strength conditions of the Rubic screen. Conditions containing concentrations of phosphate ions of 100 mM or above are shown in dark orange, while all other cells containing phosphate ions are shown in light orange. Correspondence of these cells with the highest T_m values again supports the stabilizing effect of phosphate ions on the EbPKK2 enzyme.

^{31}P-NMR chain length determination of sodium polyphosphate

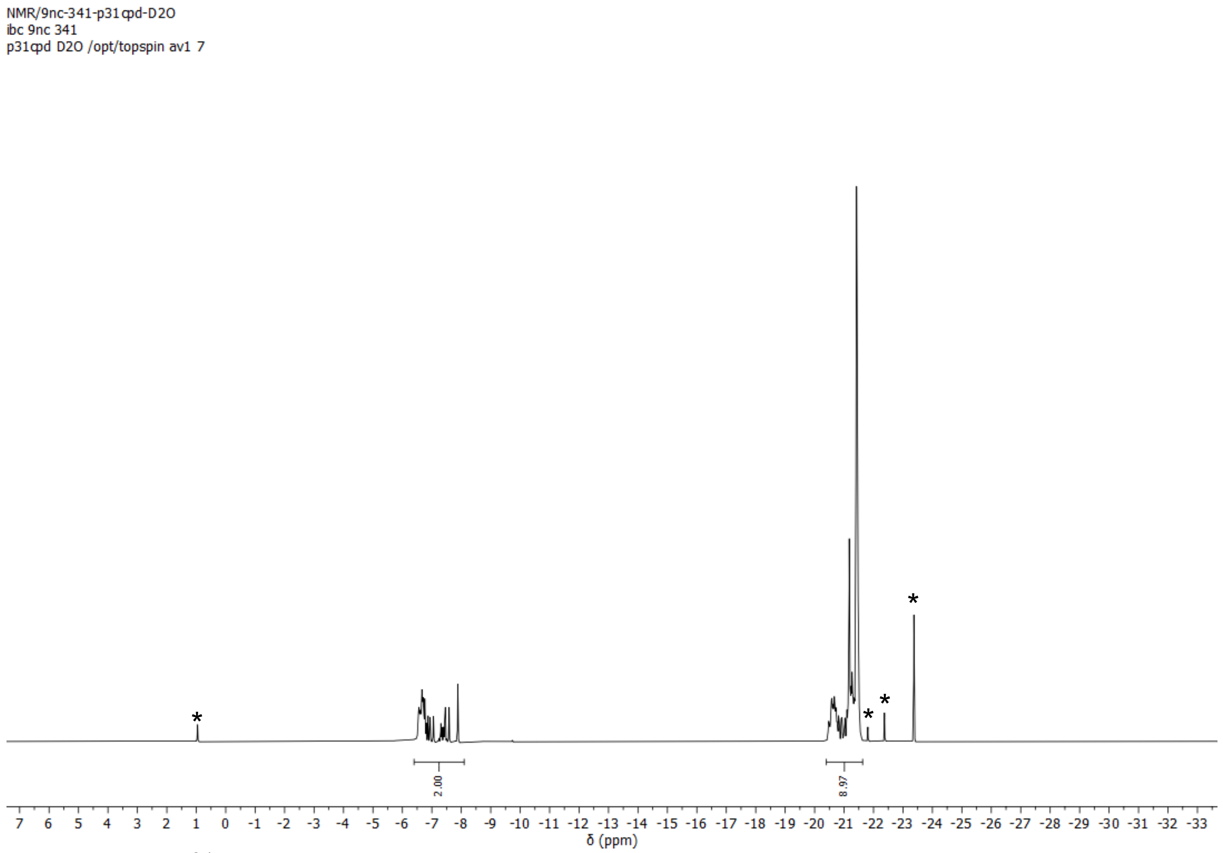

Figure S24: ^{31}P-NMR spectrum of sodium hexametaphosphate crystals, +200 mesh 96 % / Graham´s salt (Sigma-Aldrich 305553). Given the heterogeneity observed in the NMR spectrum, we used the method of Lindner et al. to determine the average chain length.[5] Terminal phosphate groups give rise to signals around -7 ppm, while internal phosphates are seen around -21 ppm. Starred peaks represent small amounts of orthophosphate (left) and cyclic phosphates (right). Setting the integral at -7 ppm to 2 (for the two termini per chain) gives an integration of 9 for the internal phosphates, indicating an average chain length of 11 overall. For further information on the assignment of ^{31}P-NMR of polyphosphates please see Christ et al.[6]

Note that many products sold as Graham´s salt or sodium hexametaphosphate show different polyphosphate chain lengths. For this product (Sigma-Aldrich 305553), the supplier states that the average chain length traditionally ranges from 9.5 to 14.5 phosphate units, which is in accordance with ^{31}P-NMR spectrum above.

LC-DAD-Q-MS analysis of AMPs (1a-10a)

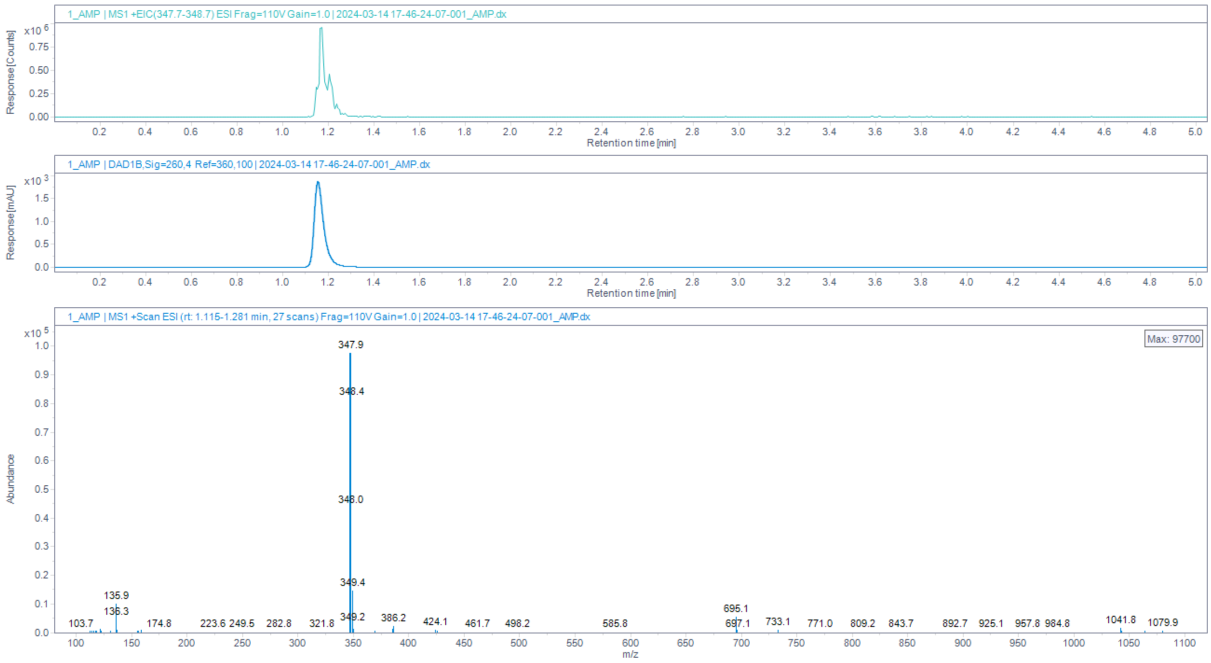

Figure S25: LC-DAD-Q-MS analysis of **1a**.

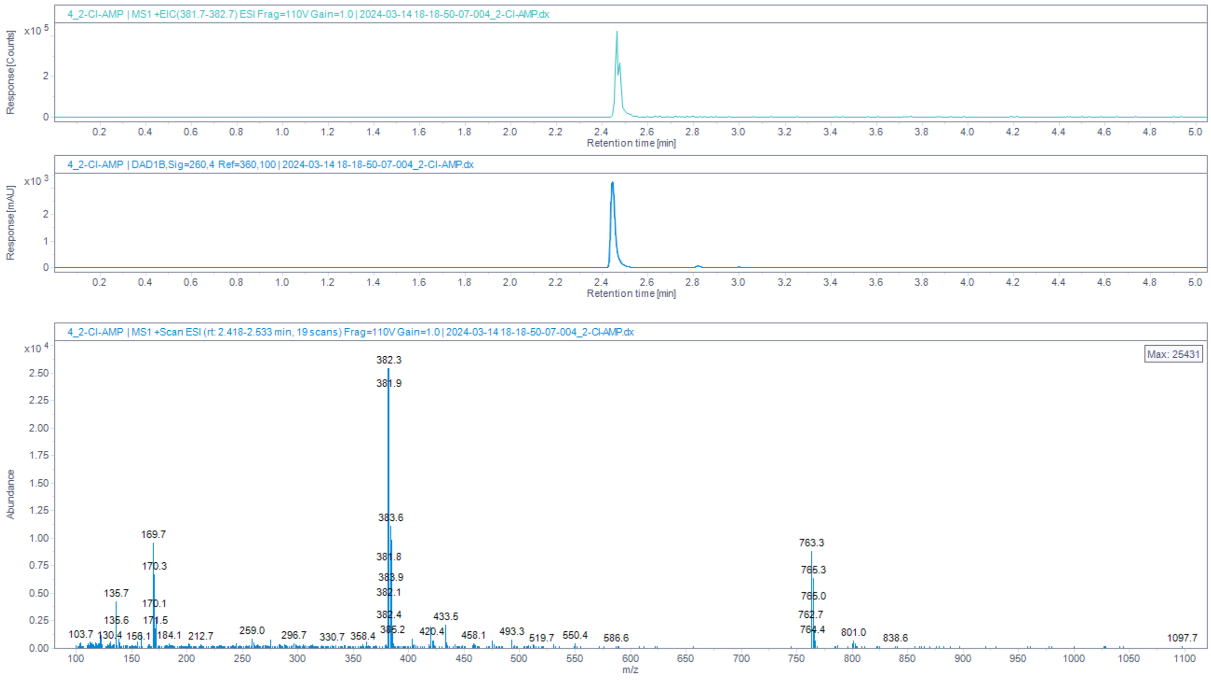

Figure S26: LC-DAD-Q-MS analysis of **2a**.

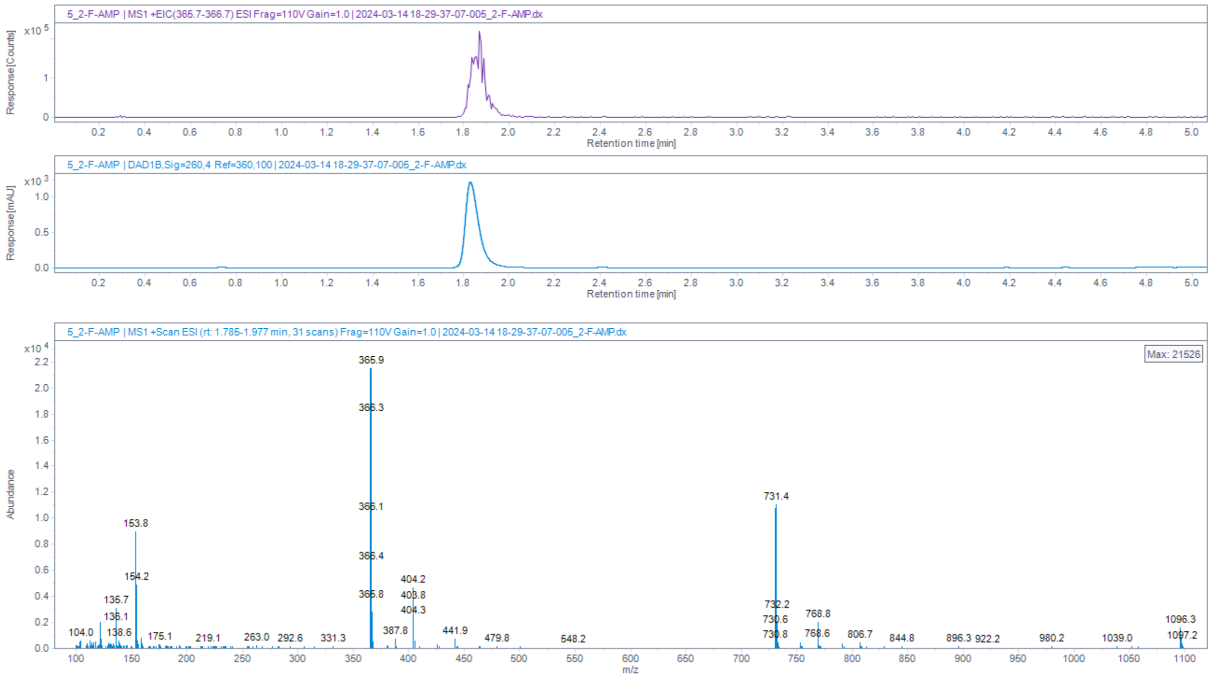

Figure S27: LC-DAD-Q-MS analysis of **3a**.

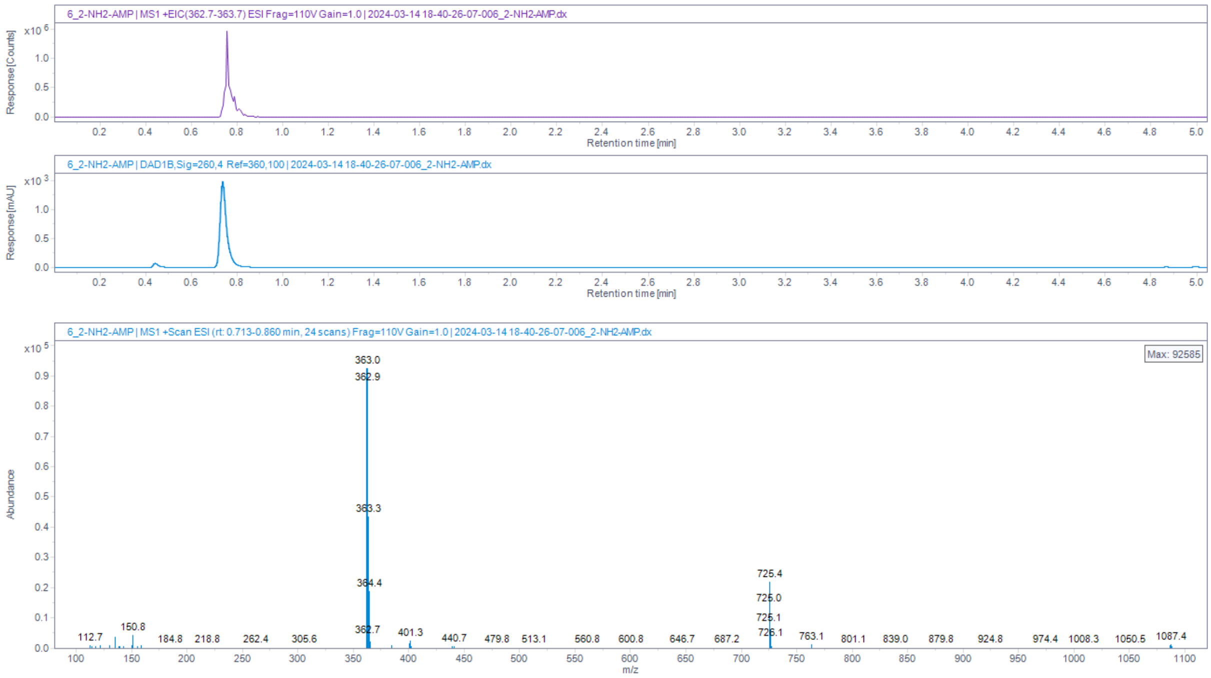

Figure S28: LC-DAD-Q-MS analysis of **4a**.

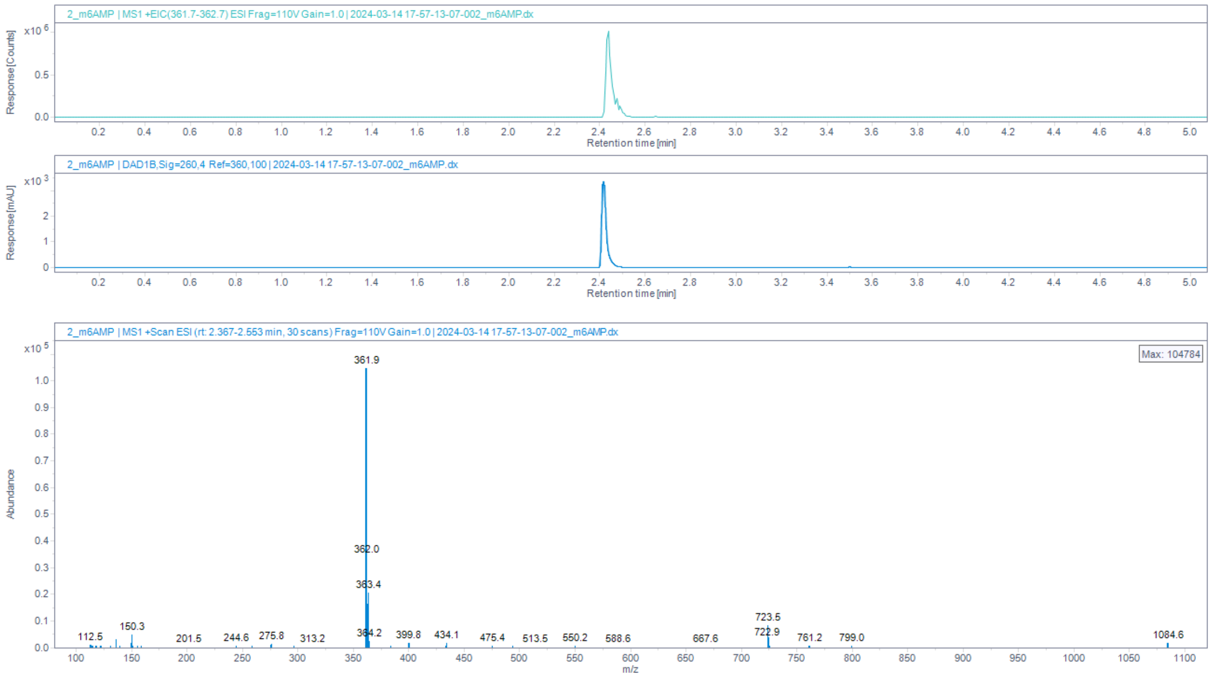

Figure S29: LC-DAD-Q-MS analysis of **5a**.

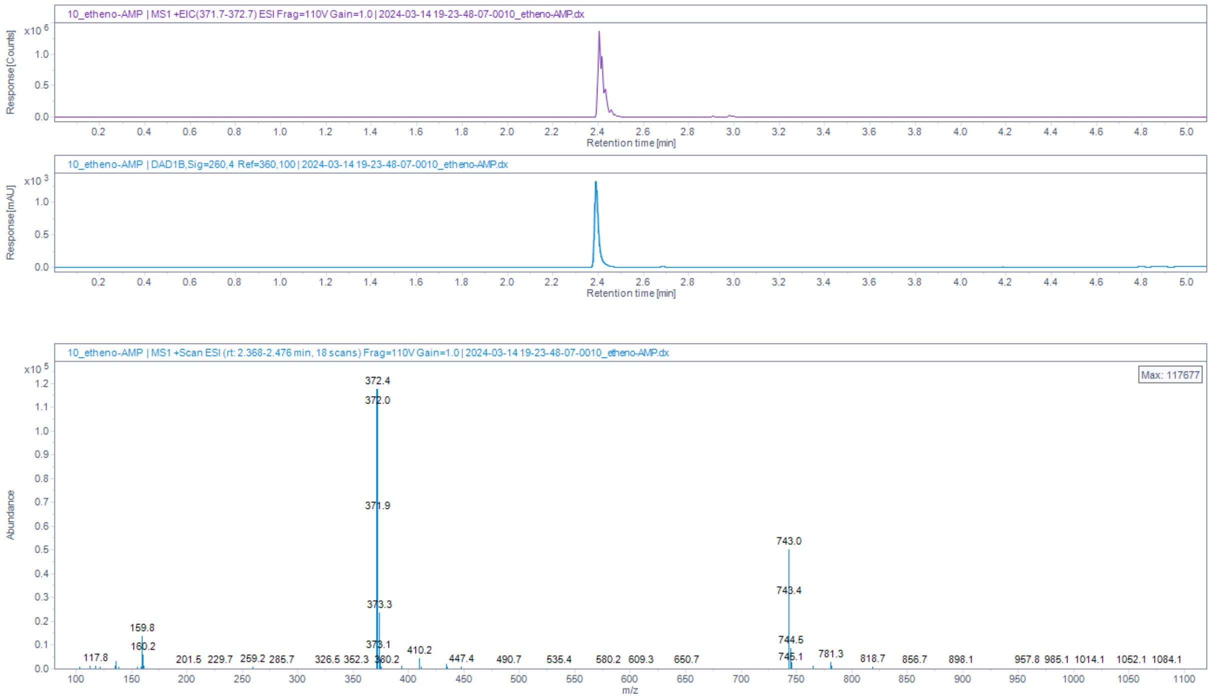

Figure S30: LC-DAD-Q-MS analysis of **6a**.

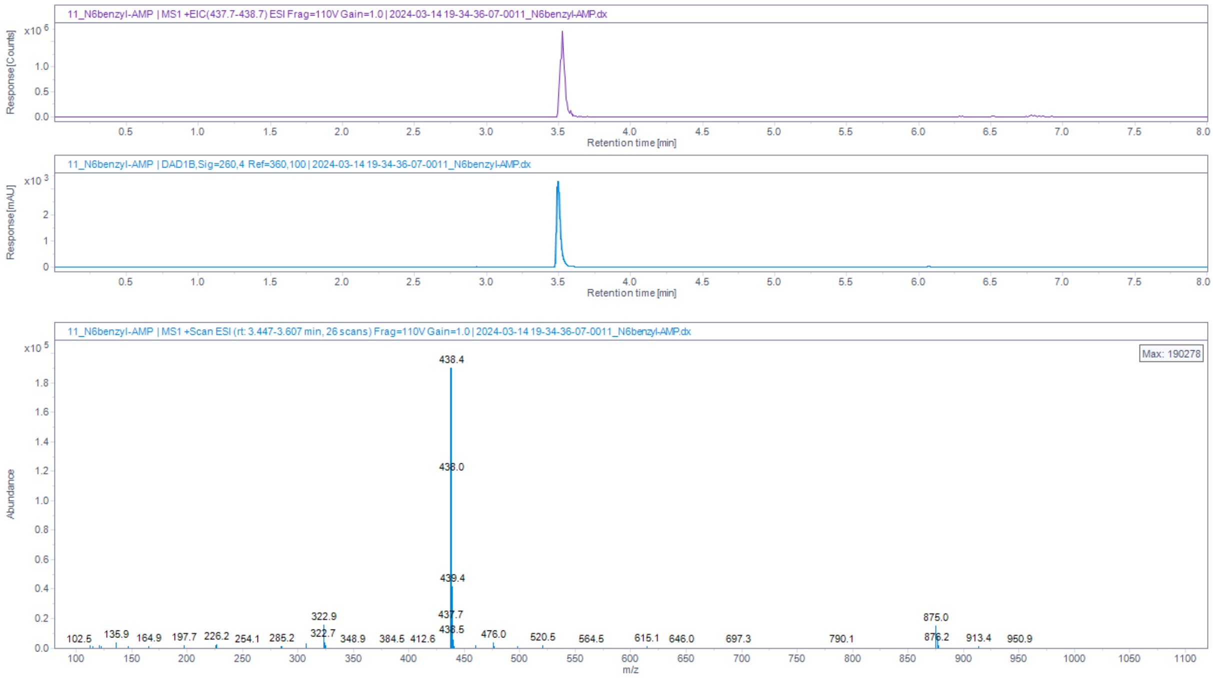

Figure S31: LC-DAD-Q-MS analysis of **7a**.

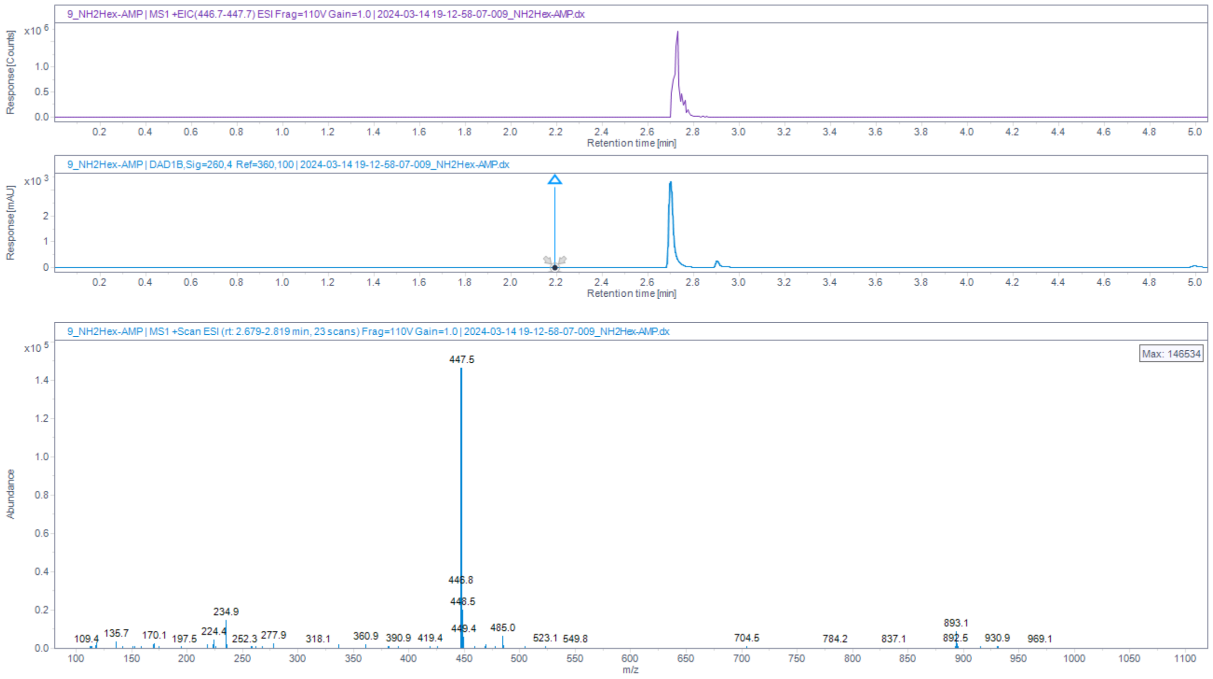

Figure S32: LC-DAD-Q-MS analysis of **8a**.

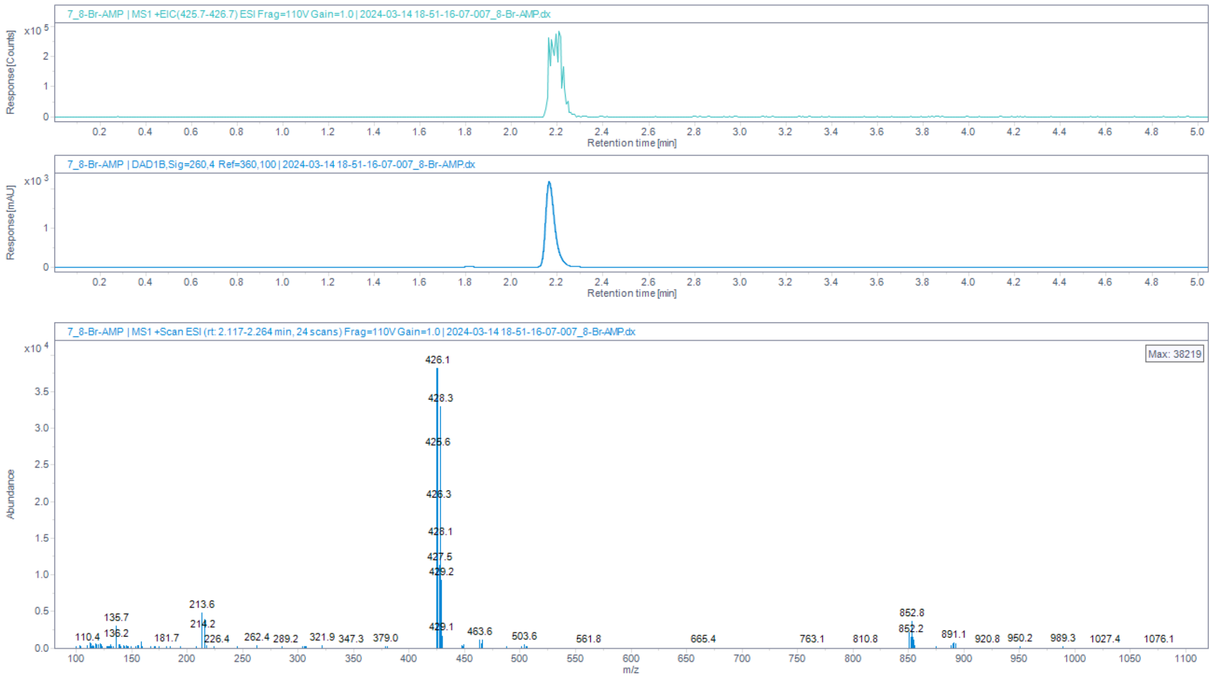

Figure S33: LC-DAD-Q-MS analysis of **9a**.

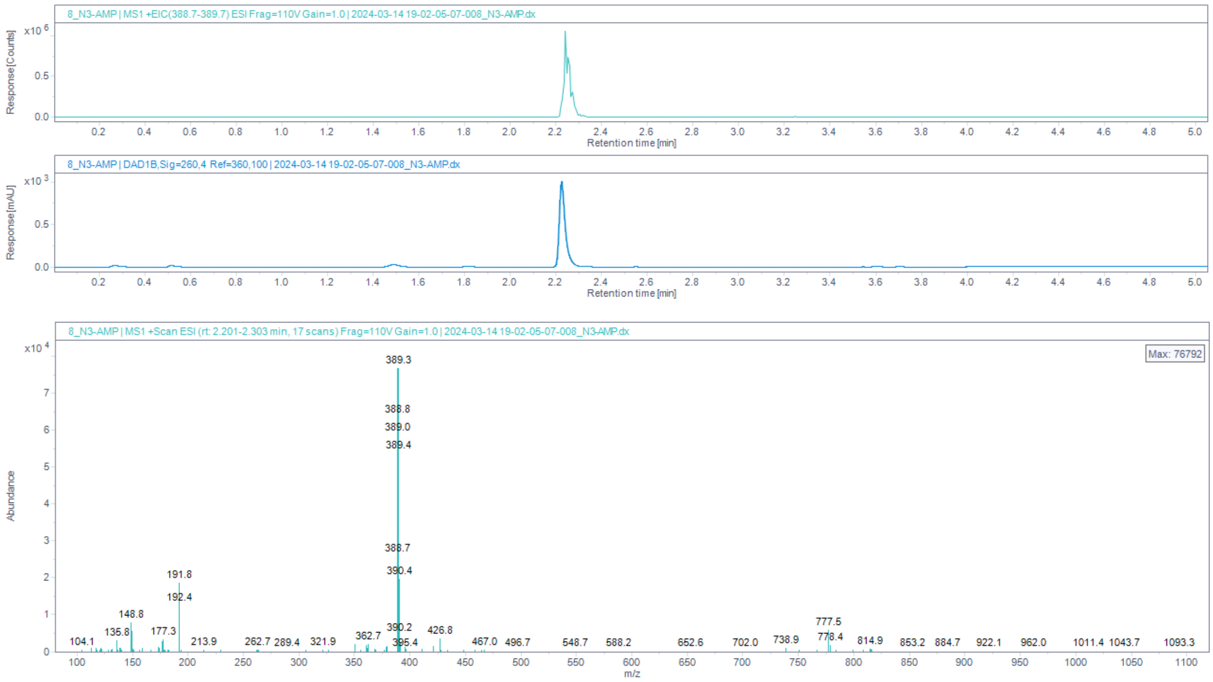

Figure S34: LC-DAD-Q-MS analysis of **10a**.

LC-DAD-Q-MS analysis of enzymatic reactions (analytical scale)

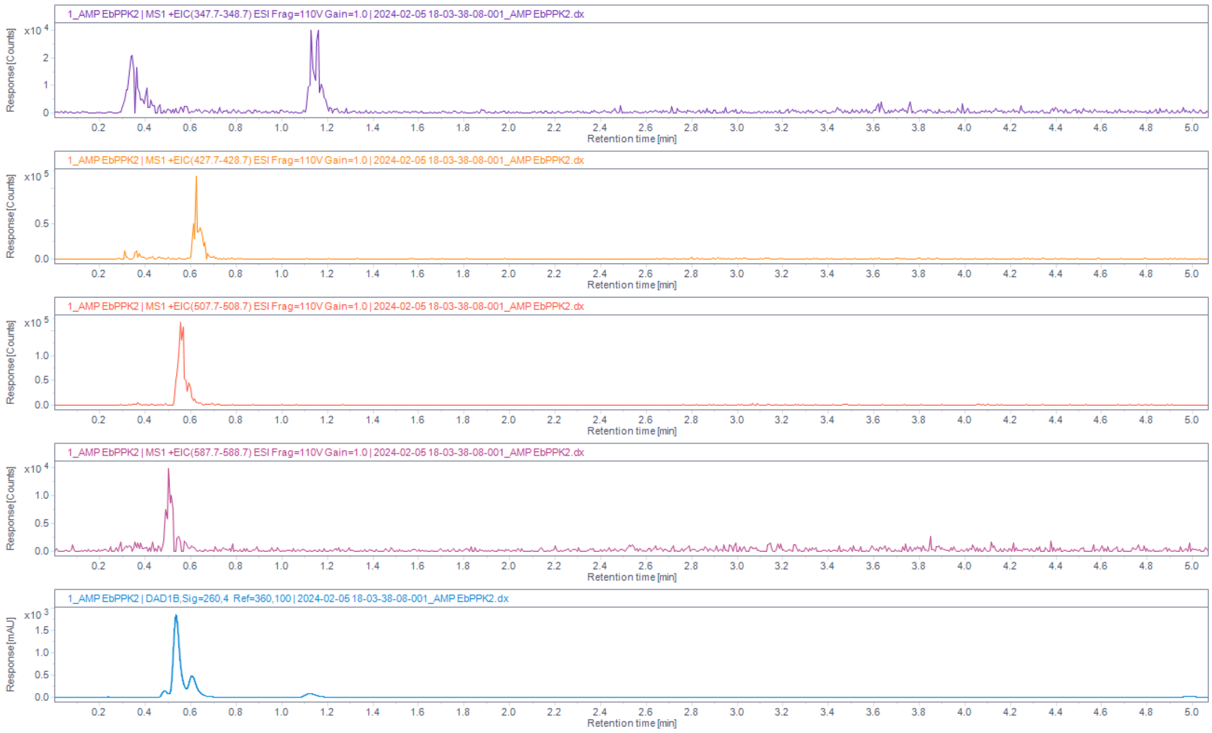

Figure S35: LC-DAD-Q-MS analysis of the EbPPK2 reaction starting from **1a**. EIC for **1a**, **1b**, **1c**, **1d** and the UV trace at 260 nm.

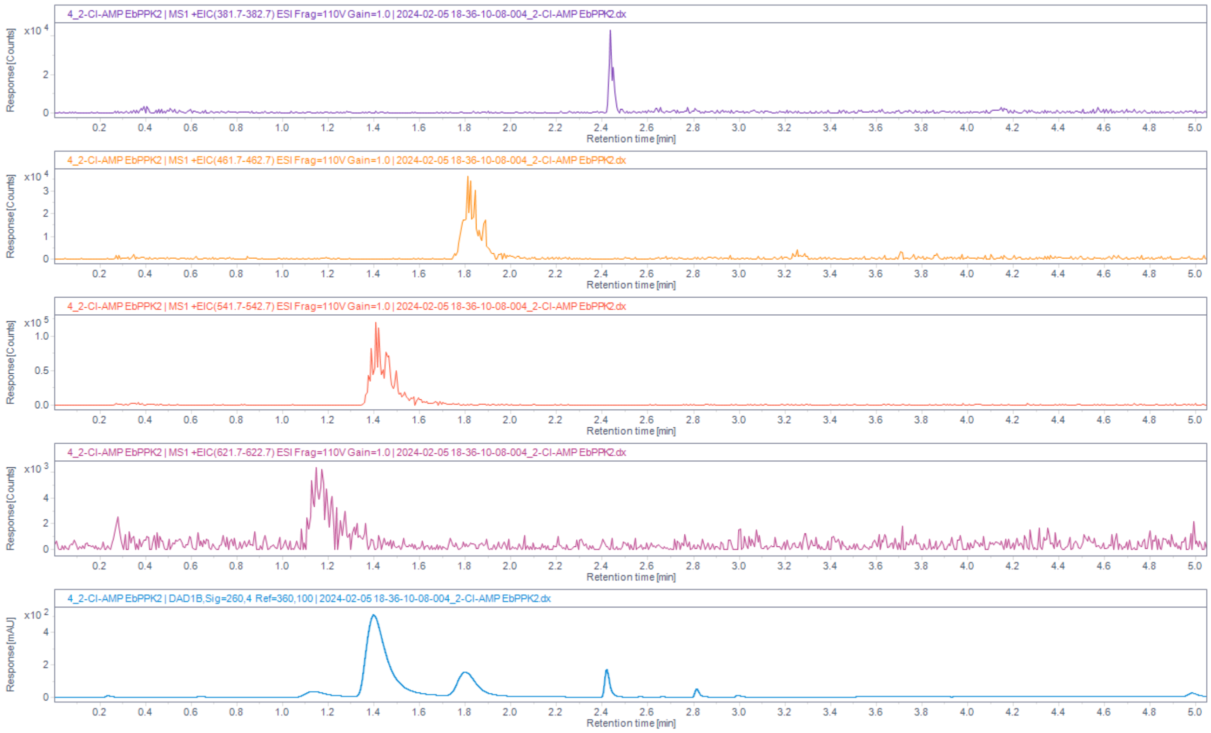

Figure S36: LC-DAD-Q-MS analysis of the EbPPK2 reaction starting from **2a**. EIC for **2a**, **2b**, **2c**, **2d** and the UV trace at 260 nm. Note that the UV signal at 2.8 min corresponds to 2-chloroadenine.

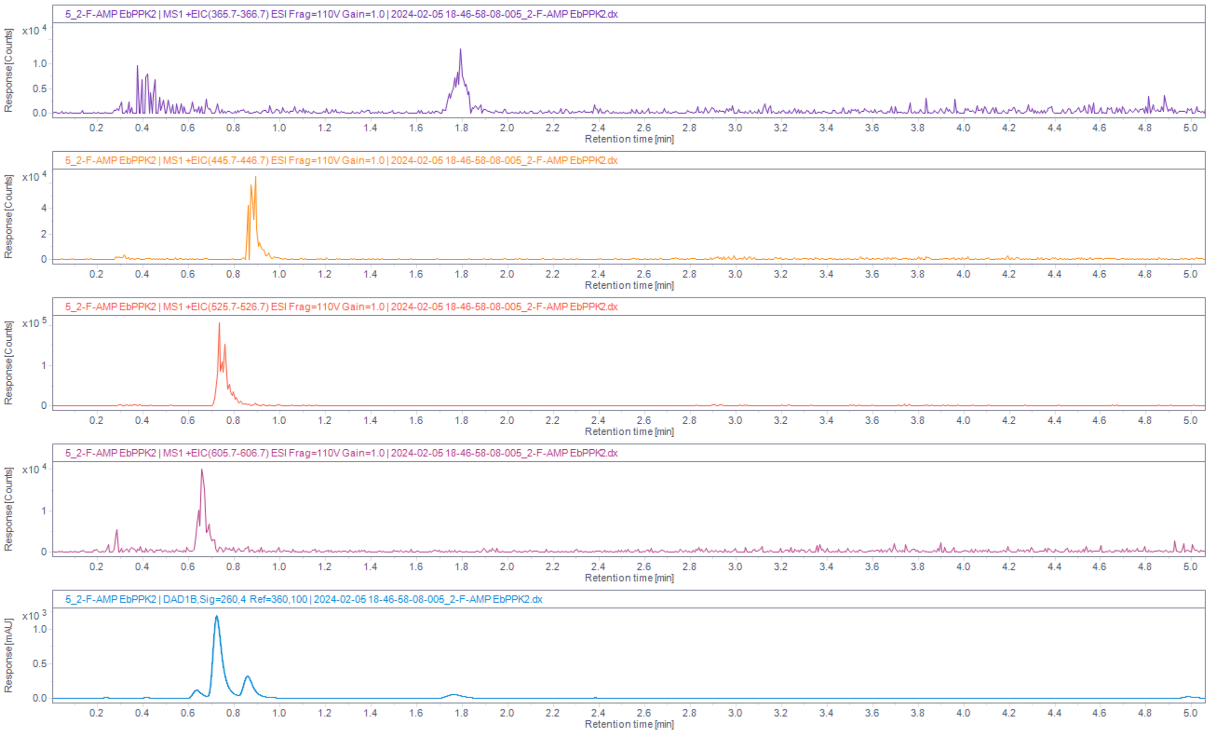

Figure S37: LC-DAD-Q-MS analysis of the EbPPK2 reaction starting from **3a**. EIC for **3a**, **3b**, **3c**, **3d** and the UV trace at 260 nm.

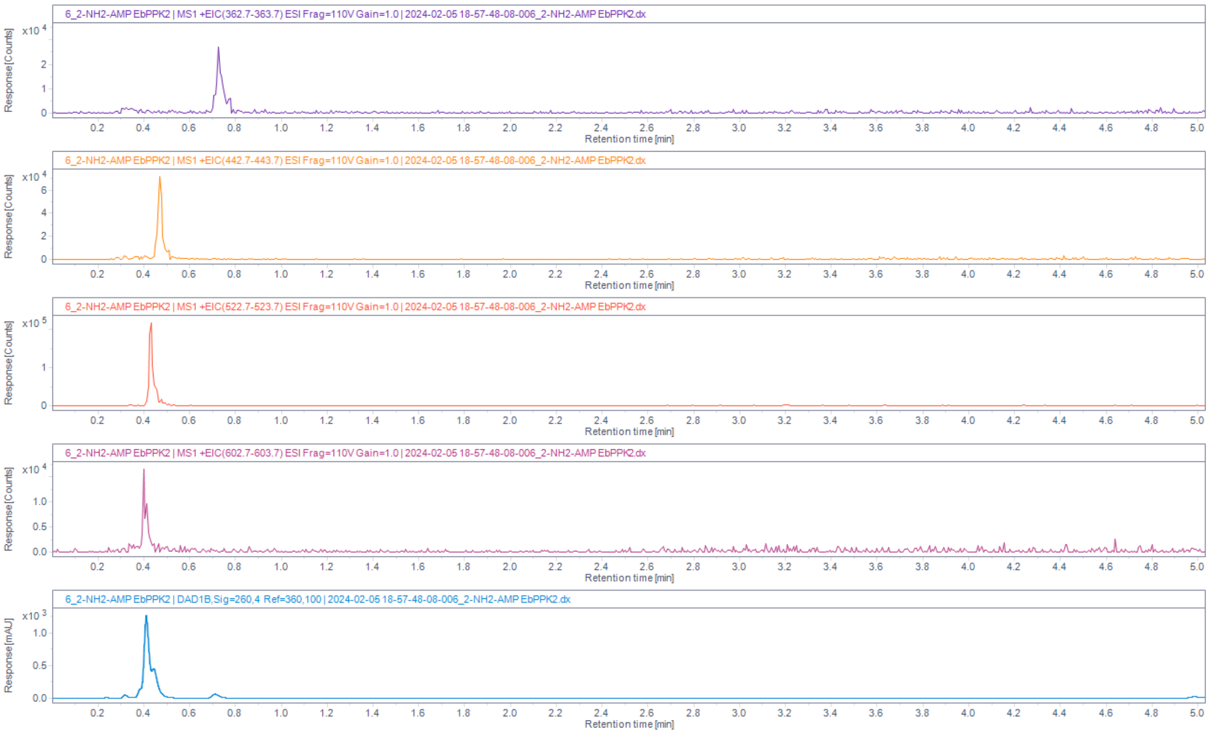

Figure S38: LC-DAD-Q-MS analysis of the EbPPK2 reaction starting from **4a**. EIC for **4a**, **4b**, **4c**, **4d** and the UV trace at 260 nm.

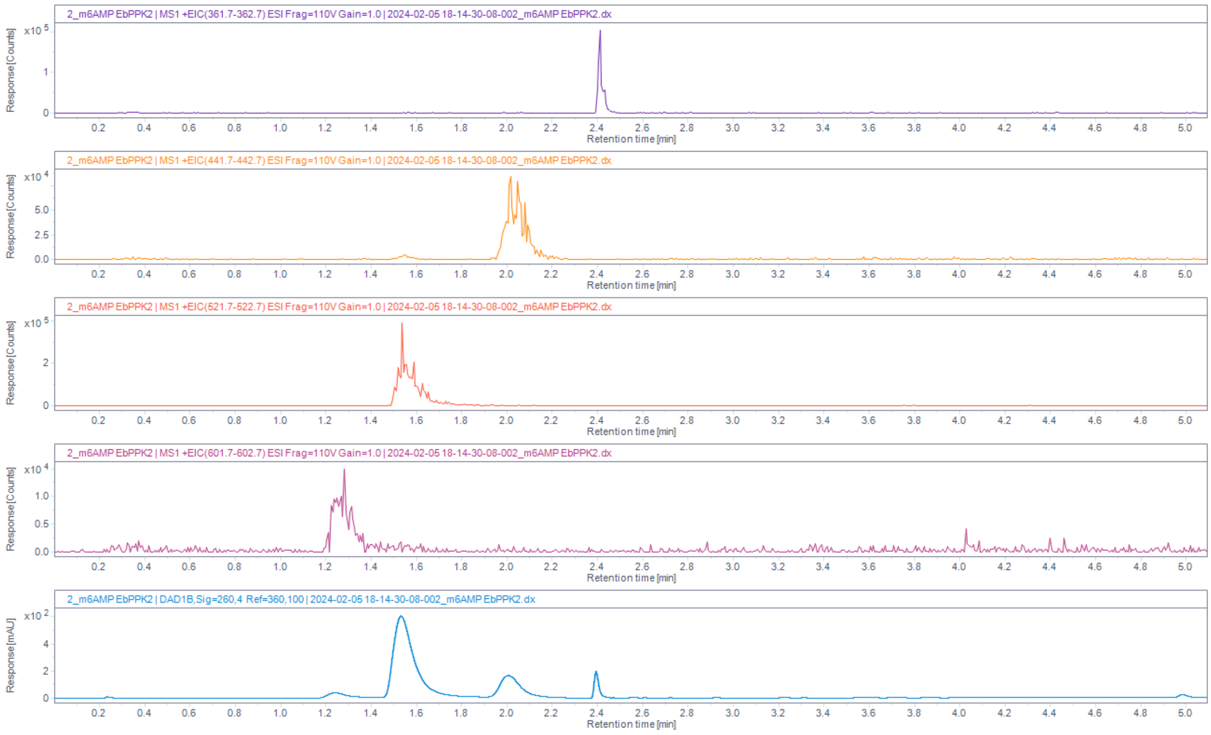

Figure S39: LC-DAD-Q-MS analysis of the EbPPK2 reaction starting from **5a**. EIC for **5a**, **5b**, **5c**, **5d** and the UV trace at 260 nm.

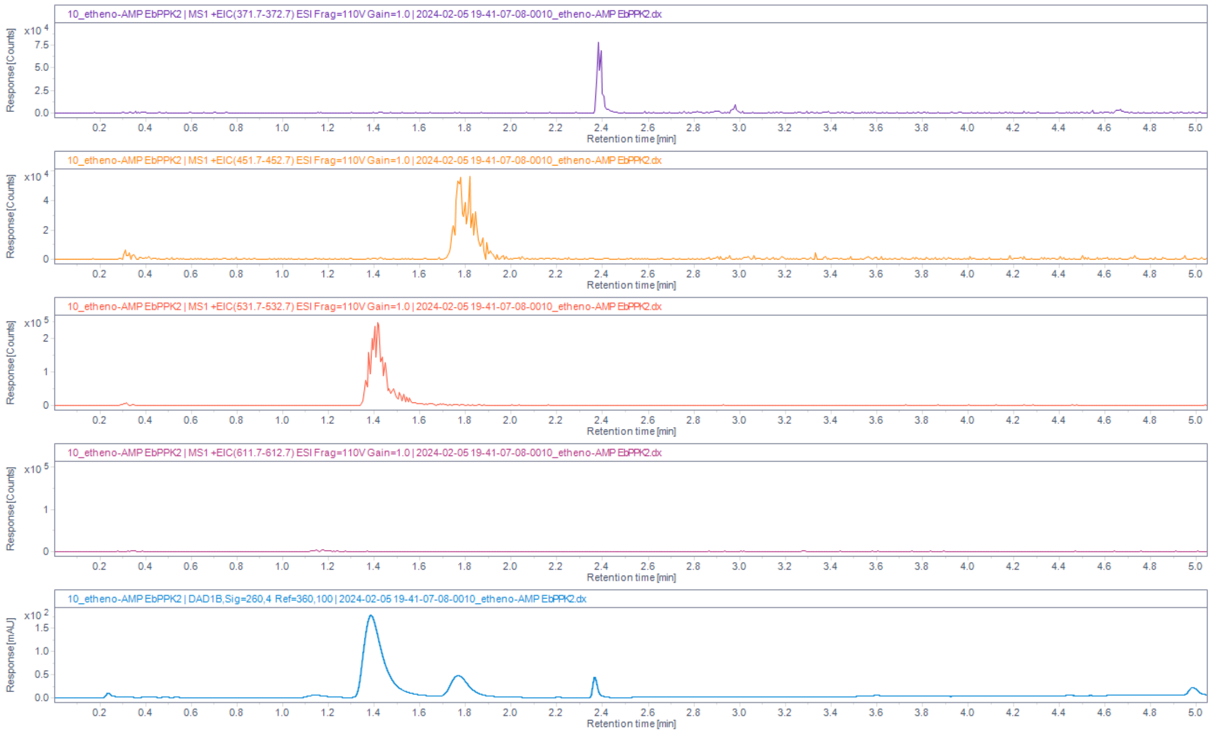

Figure S40: LC-DAD-Q-MS analysis of the EbPPK2 reaction starting from **6a**. EIC for **6a**, **6b**, **6c**, **6d** and the UV trace at 260 nm.

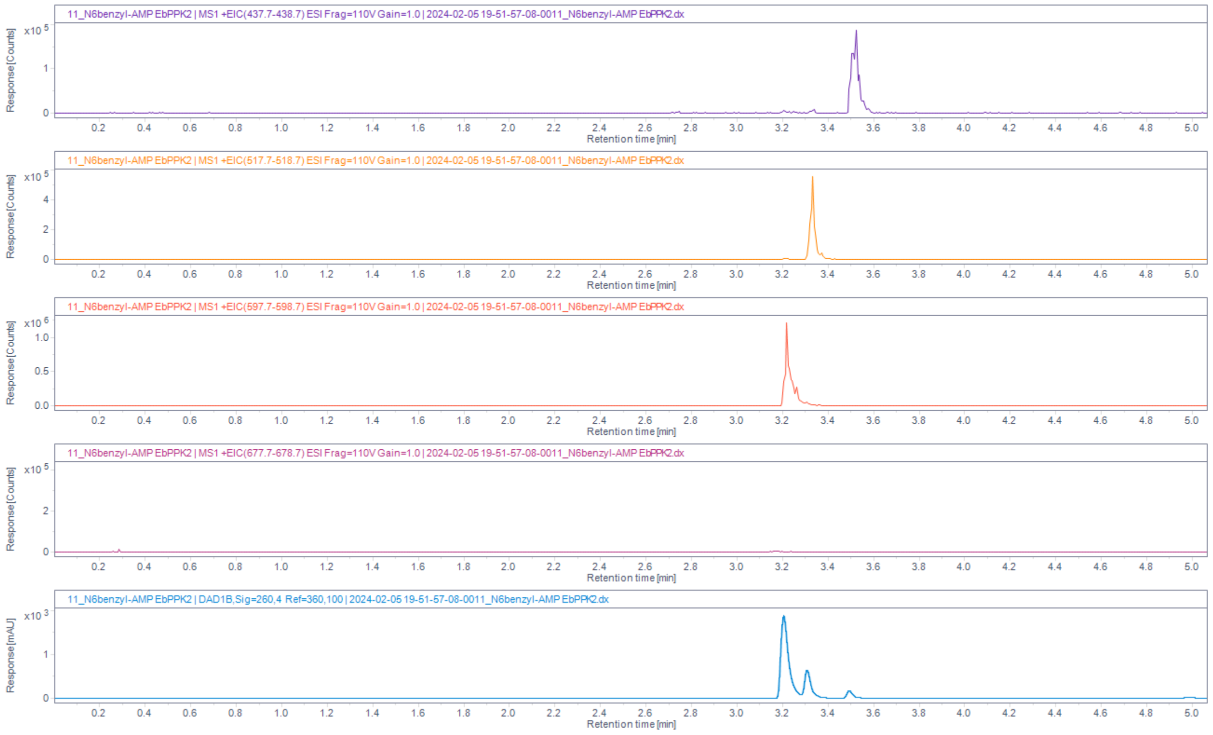

Figure S41: LC-DAD-Q-MS analysis of the EbPPK2 reaction starting from **7a**. EIC for **7a**, **7b**, **7c**, **7d** and the UV trace at 260 nm.

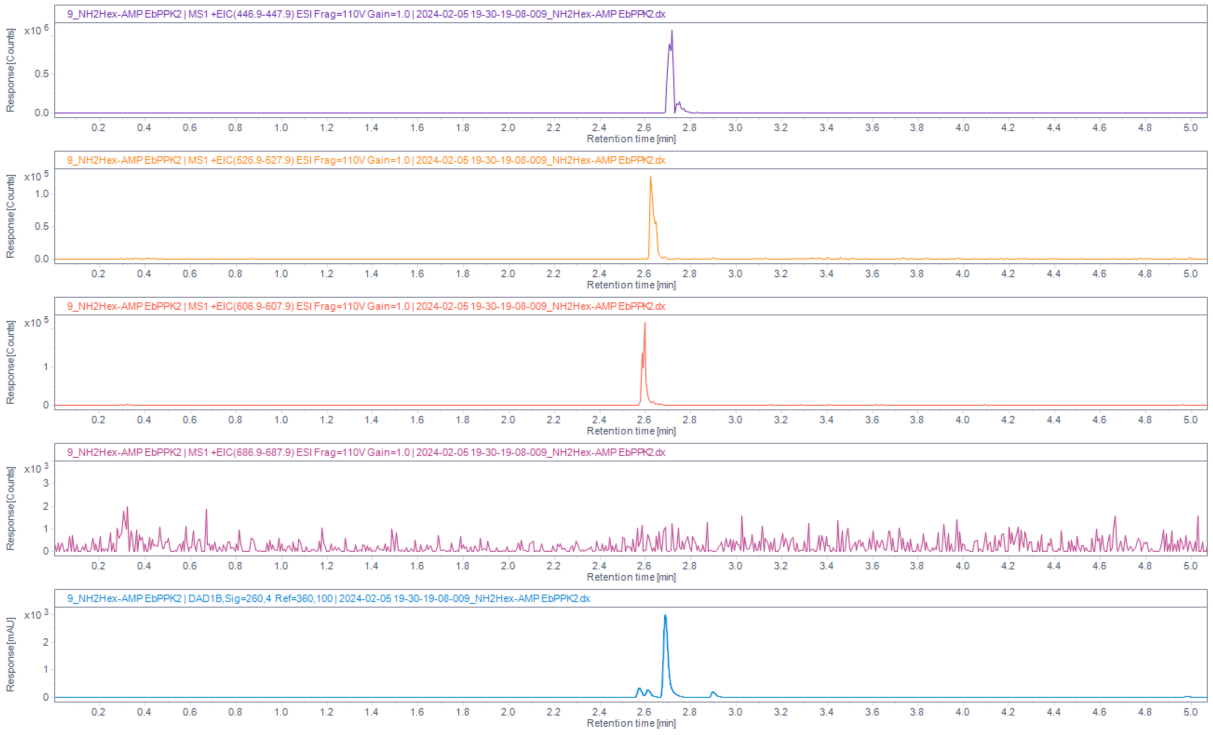

Figure S42: LC-DAD-Q-MS analysis of the EbPPK2 reaction starting from **8a**. EIC for **8a**, **8b**, **8c**, **8d** and the UV trace at 260 nm.

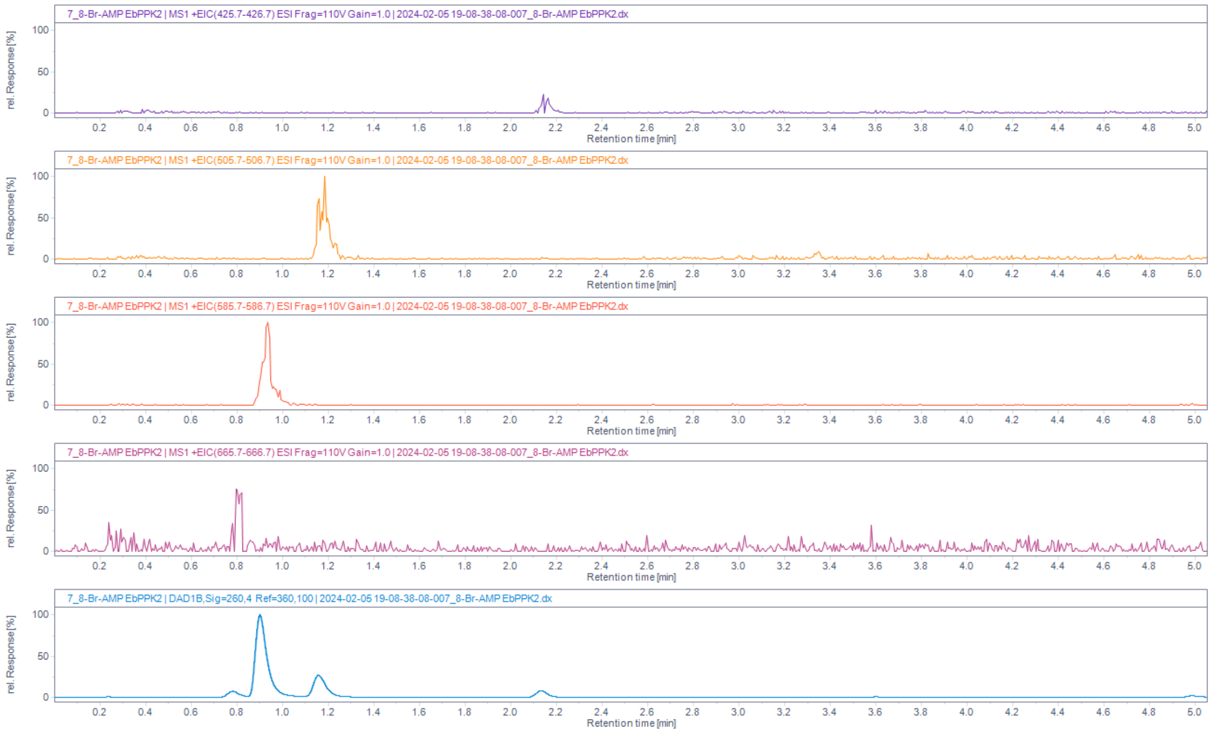

Figure S43: LC-DAD-Q-MS analysis of the EbPPK2 reaction starting from **9a**. EIC for **9a**, **9b**, **9c**, **9d** and the UV trace at 260 nm.

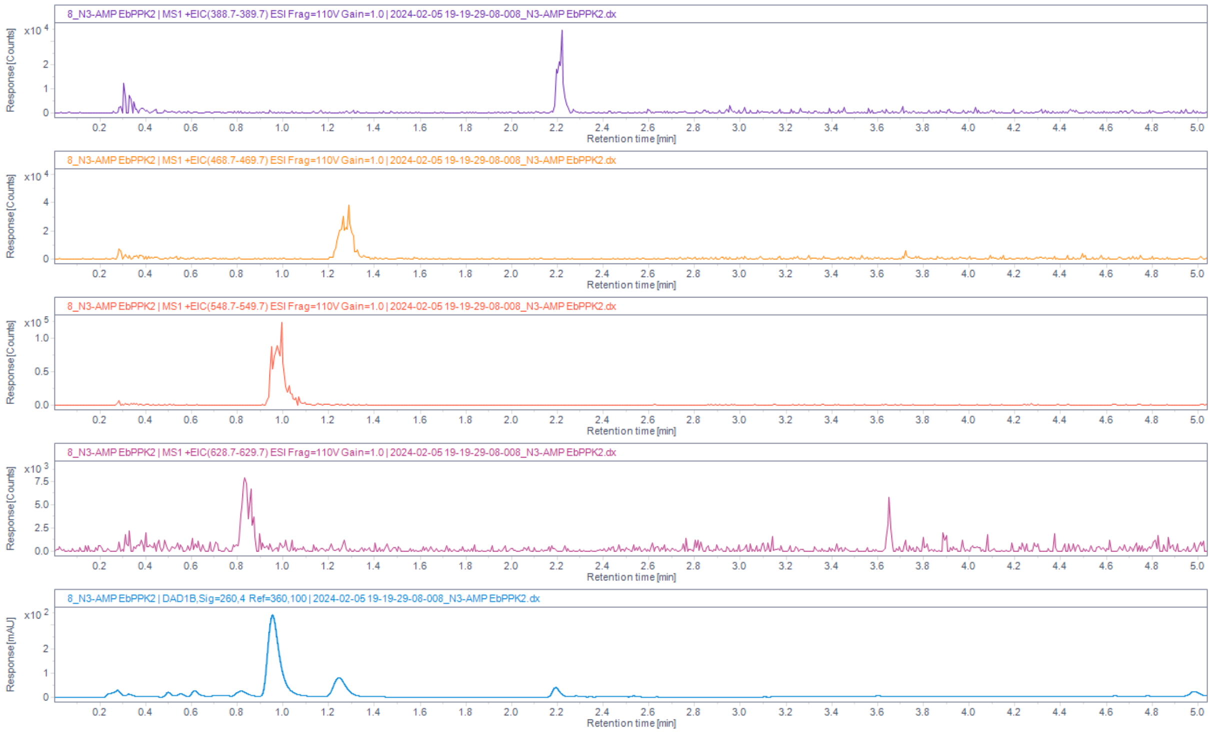

Figure S44: LC-DAD-Q-MS analysis of the EbPPK2 reaction starting from **10a**. EIC for **10a**, **10b**, **10c**, **10d** and the UV trace at 260 nm.

Purification of preparative EbPPK2 reactions

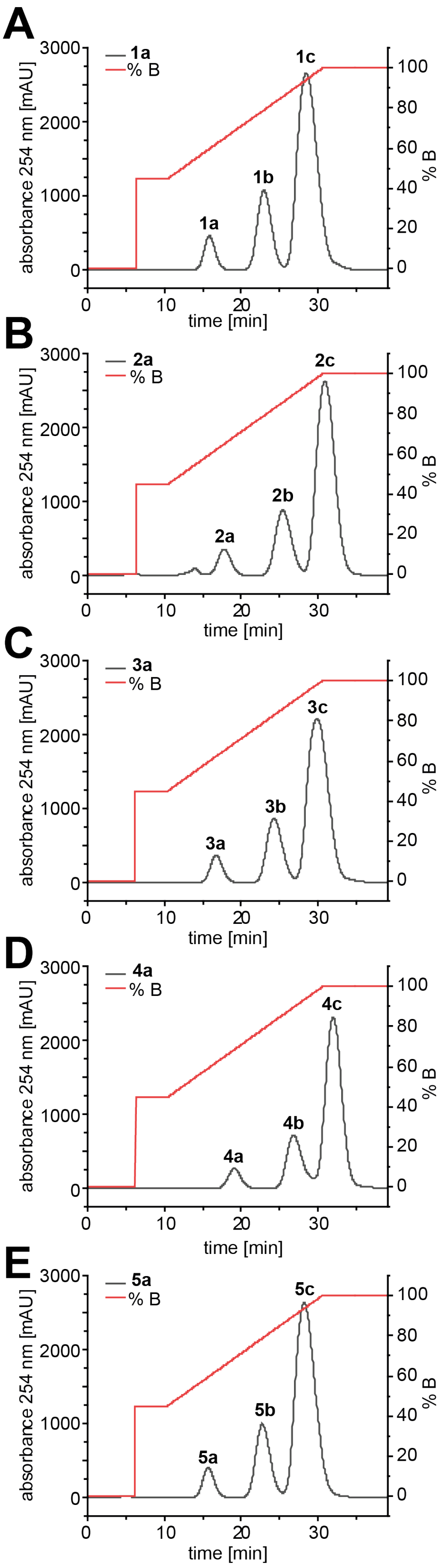

Figure S45: Preparative EbPPK2 reactions (10 µmol) purification. A)-E Chromatogram of the anion exchange chromatography performed on an ÄKTA purifier (Panel A is identical with Fig. 3A in the main text). Buffer A: ddH_2O; Buffer B: 100 mM $NaClO_4$ (adjusted to pH 4.2 with 1 M $HClO_4$).

LC-DAD-Q-MS analysis of purified ATP analogues (1c – 5c)

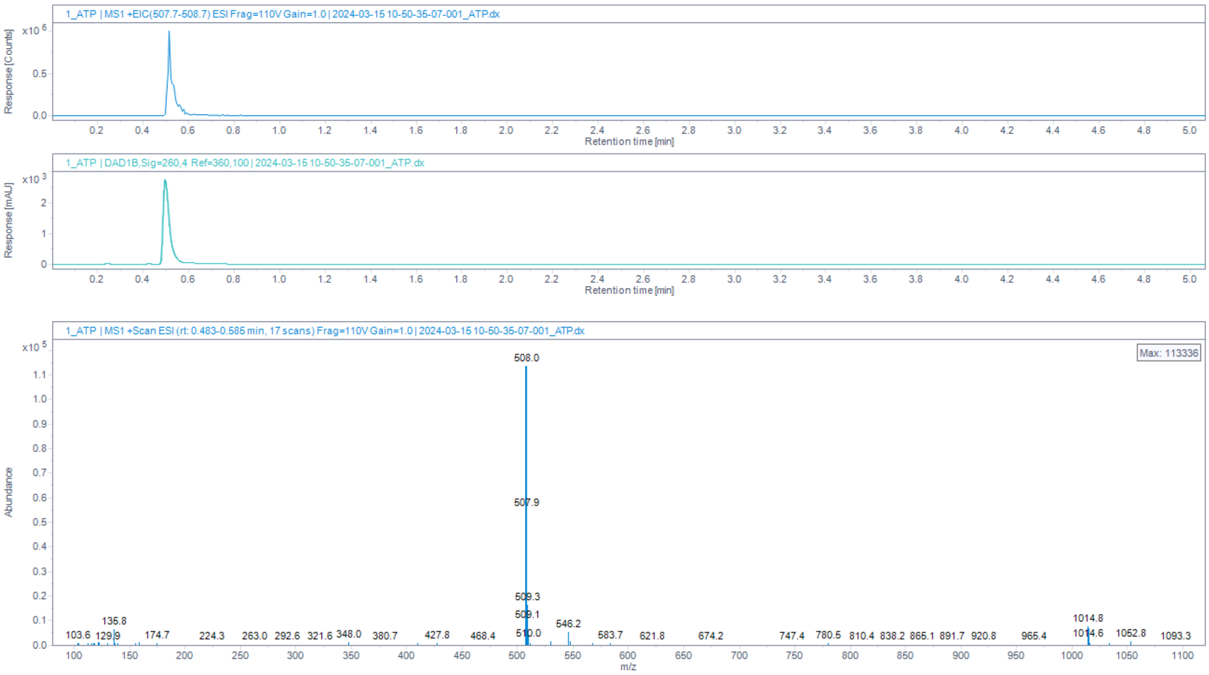

Figure S46: LC-DAD-Q-MS analysis of the purified ATP **1c**.

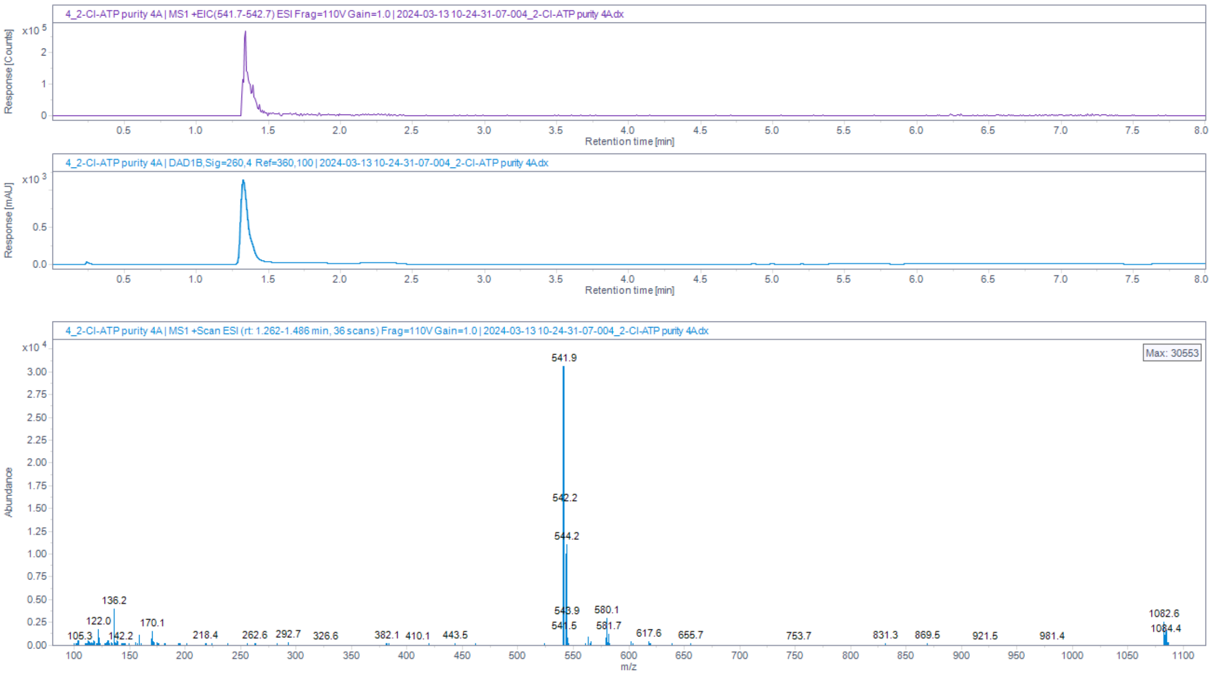

Figure S47: LC-DAD-Q-MS analysis of the purified ATP analogue **2c**.

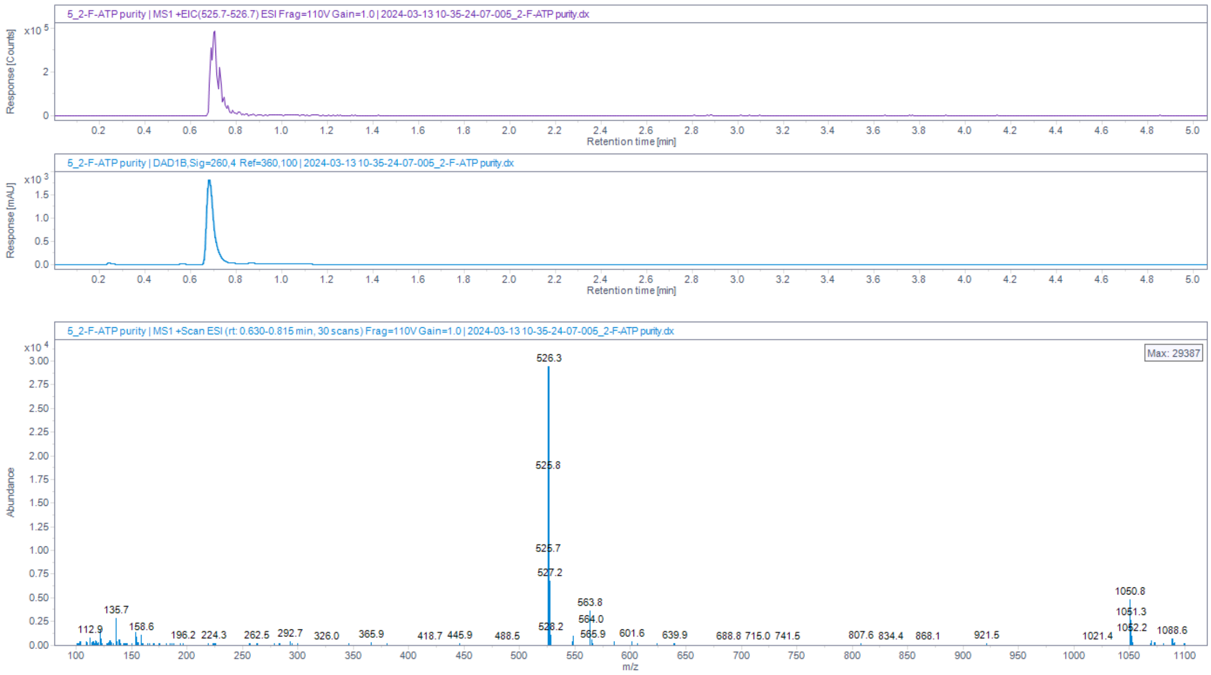

Figure S48: LC-DAD-Q-MS analysis of the purified ATP analogue **3c**.

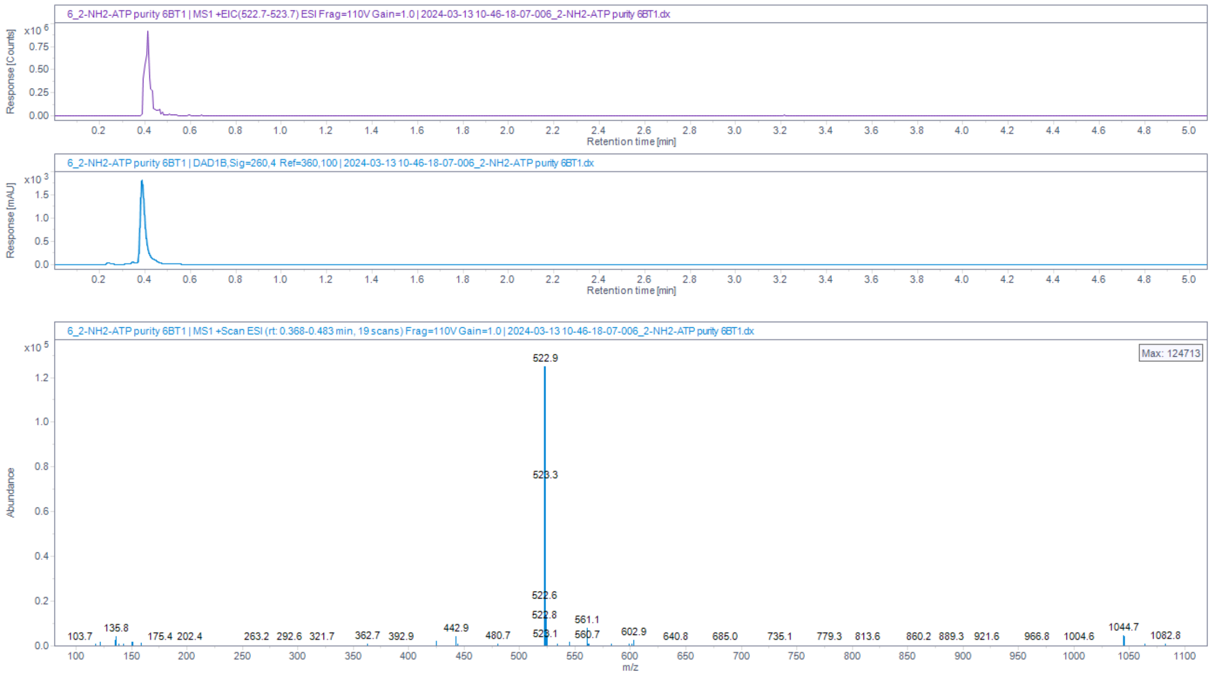

Figure S49: LC-DAD-Q-MS analysis of the purified ATP analogue **4c**.

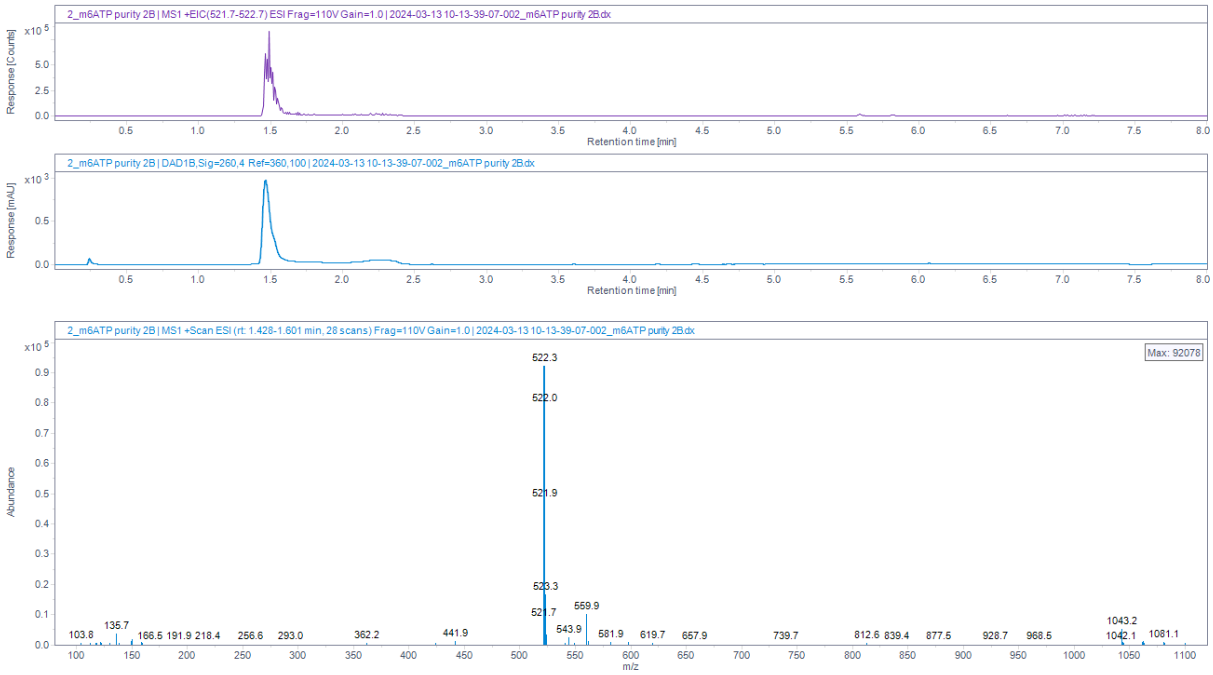

Figure S50: LC-DAD-Q-MS analysis of the purified ATP analogue **5c**.

Denaturing urea polyacrylamide gels of poly(A) polymerase reactions

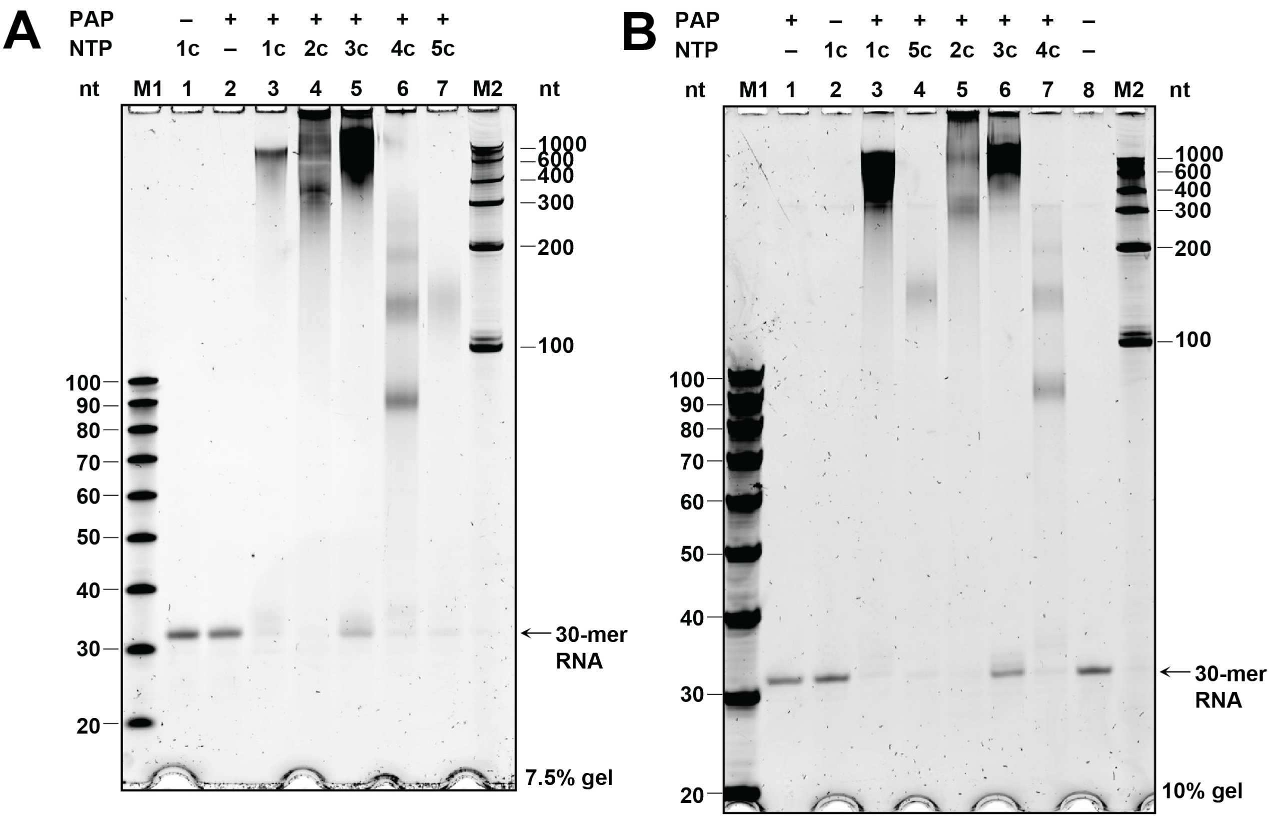

Figure S51: Denaturing urea polyacrylamide gels of the polyadenylation reactions using ScPAP and the synthesized ATP derivatives **1c** – **5c**. Replicates of Figure 4b. RNA separation was performed for 1 h at 25 – 30 W, and gels were stained with SYBR Gold and visualised with a Typhoon scanner. M1: ssDNA Oligo Length standard 20/100 (Integrated DNA Technologies); M2: RiboRuler RNA Ladder Low Range (Thermo Fisher Scientific).

[1] E. F. Pettersen, T. D. Goddard, C. C. Huang, E. C. Meng, G. S. Couch, T. I. Croll, J. H. Morris and T. E. Ferrin, *Protein Science* **2021**, *30*, 70-82.
[2] E. Krieger, K. Joo, J. Lee, J. Lee, S. Raman, J. Thompson, M. Tyka, D. Baker and K. Karplus, *Proteins: Structure, Function, and Bioinformatics* **2009**, *77*, 114-122.
[3] F. H. Niesen, H. Berglund and M. Vedadi, *Nature Protocols* **2007**, *2*, 2212-2221.
[4] J. Jumper, R. Evans, A. Pritzel, T. Green, M. Figurnov, O. Ronneberger, K. Tunyasuvunakool, R. Bates, A. Žídek, A. Potapenko, A. Bridgland, C. Meyer, S. A. A. Kohl, A. J. Ballard, A. Cowie, B. Romera-Paredes, S. Nikolov, R. Jain, J. Adler, T. Back, S. Petersen, D. Reiman, E. Clancy, M. Zielinski, M. Steinegger, M. Pacholska, T. Berghammer, S. Bodenstein, D. Silver, O. Vinyals, A. W. Senior, K. Kavukcuoglu, P. Kohli and D. Hassabis, *Nature* **2021**, *596*, 583-589.
[5] S. N. Lindner, D. Vidaurre, S. Willbold, S. M. Schoberth and V. F. Wendisch, *Appl Environ Microbiol* **2007**, *73*, 5026-5033.
[6] J. J. Christ, S. Willbold and L. M. Blank, *Analytical Chemistry* **2019**, *91*, 7654-7661.
